# Southward growth of Mauna Loa’s dike-like magma body driven by topographic stress

**DOI:** 10.1038/s41598-021-89203-6

**Published:** 2021-05-10

**Authors:** Bhuvan Varugu, Falk Amelung

**Affiliations:** Rosenstiel School of Marine and Atmospheric Sciences, University of Miami, 4600 Rickenbacker Causeway, Miami, FL 33149 USA

**Keywords:** Geophysics, Volcanology

## Abstract

Space-geodetic observations of a new period of inflation at Mauna Loa volcano, Hawaii, recorded an influx of 0.11 km^3^ of new magma into it’s dike-like magma body during 2014–2020. The intrusion started after at least 4 years of decollement slip under the eastern flank creating > 0.15 MPa opening stresses in the rift zone favorable for magma intrusion. Volcanoes commonly respond to magma pressure increase with the injection of a dike, but Mauna Loa responded with lateral growth of its magma body in the direction of decreasing topographic stress. In 2017, deformation migrated back, and inflation continued at the pre-2015 location. Geodetic inversions reveal a 8 × 8.5, 10 × 3 and 9 × 4 km^2^ dike-like magma body during the 2014–2015, 2015–2018 and 2018–2020 periods, respectively, and an average decollement slip of ~ 23 cm/year along a 10 × 5 km^2^ fault. The evolution of the dike-like magma body including the reduction in vertical extent is consistent with a slowly ascending dike propagating laterally when encountering a stress barrier and freezing its tip when magma influx waned. Overall, the magma body widened about 4.5 m during 2002–2020.

## Introduction

One of the most significant advances in volcanology in the past decade are observations showing that the subsurface accumulation and migration of magma follows gradients in the stress field, following concepts of dike emplacement and growth put forward by Anderson^1^, Delaney et al.^2^ and Rubin^3^. Examples include, the encouragement and suppression of shallow magma accumulation under collapse calderas and volcanic edifices, respectively^4–6^, the alignment of propagating dikes to the regional stress field immediately after dike injection^7^, twisting of propagating sills to dikes due to unloading stresses from caldera collapse^5^, in some settings widening of dikes in areas of reduced loading stress because of low topography^8^‚ and dike intrusions following stress gradients due to previous intrusions and earthquakes^9,10^. Yet these studies were conducted after eruptions. Here we demonstrate that Mauna Loa’s dike-like magma body evolves in response to changes in the pressurization rate, and that this response is shaped by topographic stresses and stress perturbations due to decollement slip, offering the opportunity to use stress field information for hazard assessment.

## The 2014–2020 unrest period

Mauna Loa, the largest volcano on Earth, grows by lava flows at the surface, by repeated magma intrusions into the southwest rift zone (SWRZ) and northeast rift zone (NERZ) and by seismic and aseismic motion along a low-angle, upward-dipping basal decollement fault under the volcanic pile^11^. There is stress feedback between rift intrusion and decollement motion, with one encouraging the other and vice versa^12^. Since the last eruption in 1984, the volcano has had at least two inflation periods^10,13^, one of them associated with deep (> 35 km) seismicity^14^. Mauna Loa’s principal hazards are lava flows. A 1950 flow reached the populated coastal area in 2–3 h after the eruption began^15^.

**Figure 1:**
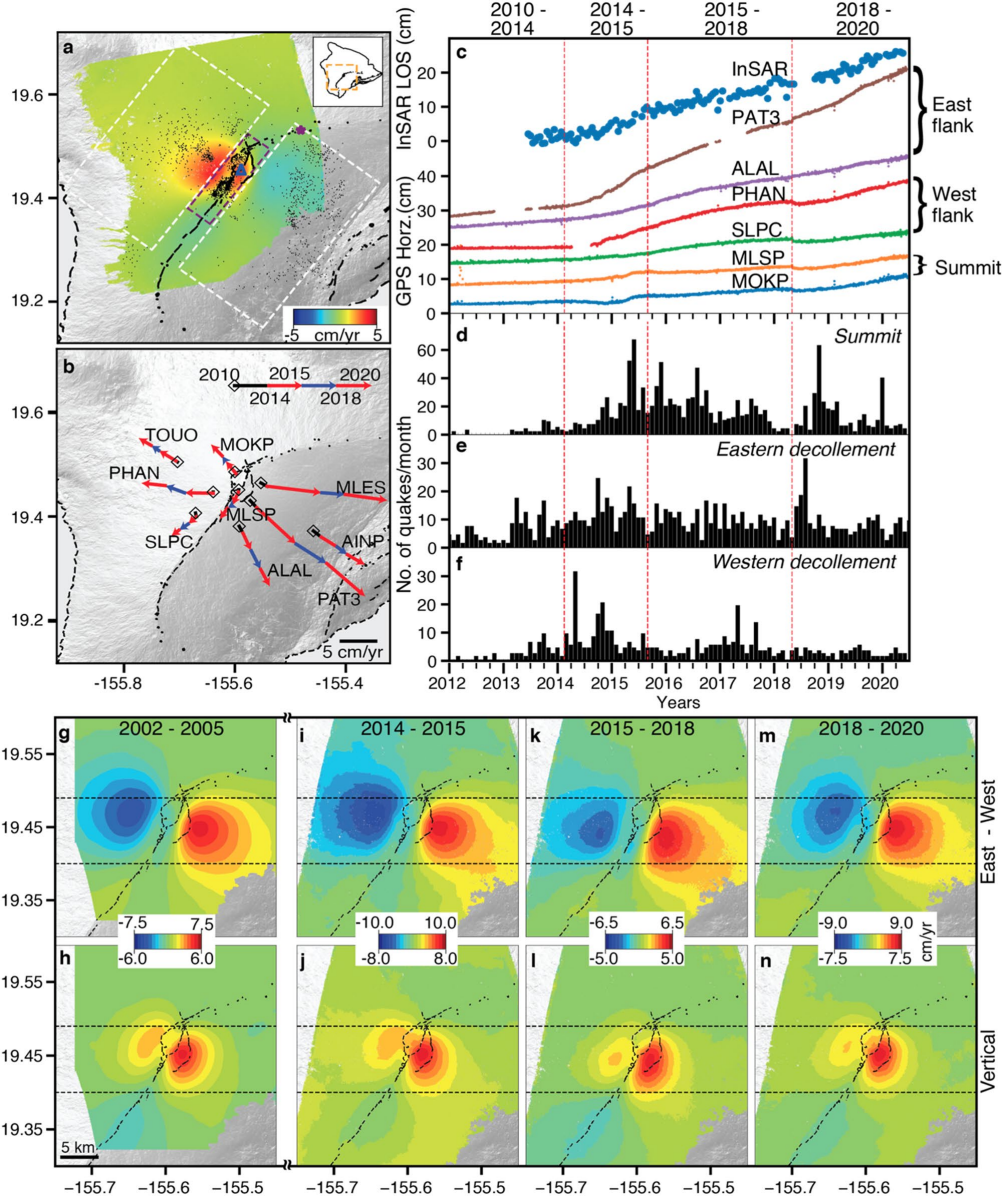
(**a**) InSAR LOS Velocity from January 2014 to May 2020 over Mauna Loa from ascending Cosmo-SkyMed imagery together with seismicity. (**b**) Cumulative GPS horizontal velocities for the 2010–2014 and the three 2014–2020 time periods. (**c**) InSAR LOS and GPS horizontal displacement time series. (**d**–**f**) monthly number of earthquakes (> M 1.0) for three sections of Mauna Loa: (**d**) under the summit (0–6 km depth), (**e**) near the eastern basal decollement (7–15 km depth), (**f**) near the western decollement fault (7–15 km depth). East–west horizontal and vertical velocities from ascending and descending InSAR during (**g**,**h**) 2002–2005, (**i**,**j**) 2014–2015, (**k**,**l**) 2015–2018, and (**m**,**n**) 2018–2020. Color scale is adjusted to enhance the shift in locus of deformation. In (**a**): white and purple rectangles: sections for seismicity counts; black dots: seismicity; purple star: reference point; blue triangle: location of InSAR timeseries in (**c**); vertical lines in (**c**–**e**): time periods discussed in paper; horizontal lines in (**g**–**n**): to highlight the southward shift of deformation during 2015–2018.

Ascending and descending Cosmo-Skymed InSAR time-series and daily GPS positions provide records about the changing deformation sources. The summit of Mauna Loa inflated during 2014–2020 at a rate of up to 6 cm/year in radar line-of-sight (LOS) direction (Fig. 1a,c, Supplementary Fig. S1.1). The horizontal displacement time series and cumulative velocities for selected GPS stations and periods show how summit deformation evolved since 2010 (Fig. 1b,c, Supplementary Fig. S1.2). During 2010–2014, the stations on the eastern flank (PAT3, ALAL) moved seaward while the stations on the western flank (PHAN, SLPC) were stable. Also, stations on the eastern and western flanks accelerated at the beginning and in mid of 2014, respectively. Stations near the caldera changed direction and velocity in August 2015 and again in 2017–2018. In the following we consider three periods separately, from January 2014 to August 2015, from August 2015 to April 2018 and from April 2018 to May 2020, referred to as time periods 1, 2 and 3 or simply as the 2014–2015, 2015–2018 and 2018–2020 periods.

The quasi-horizontal and quasi-vertical velocities inferred from the combination of ascending and descending InSAR data shows nearly identical deformation patterns for the three periods (Fig. 1g–n), but during 2015–2018 the deforming area is located ~ 2.3 km down the rift zone (Fig. 1k,l). The inflation rate decreased in 2015 but increased again in 2018 [east velocities up to 10, 6.5 and 9 cm/year for the three periods, respectively (Fig. 1i,k,m)]. The 2002–2005 period shows the same deformation pattern as during 2014–2015 but at a lower rate (east velocity up to 7.5 cm/year). The GPS positions show that the 2015 change occurred over days to weeks (see pseudo-position plot Supplementary Fig. S1.3) but don’t constrain the timing of the 2017–2018 transition. The InSAR data suggest that the inflation center migrated progressively northwards (see Supplementary Fig. S1.4).

The unrest period started already in early 2013 with a rise in seismicity on or near the eastern decollement fault (Fig. 1e). The shallow summit seismicity (0–6 km depth) increased in late 2014 (Fig. 1d) with notable peaks around May 2015 and May 2018, clustered south of the caldera. Along the western decollement fault, the seismicity is significantly less compared to the eastern decollement (Fig. 1e,f).

## Source models

**Figure 2:**
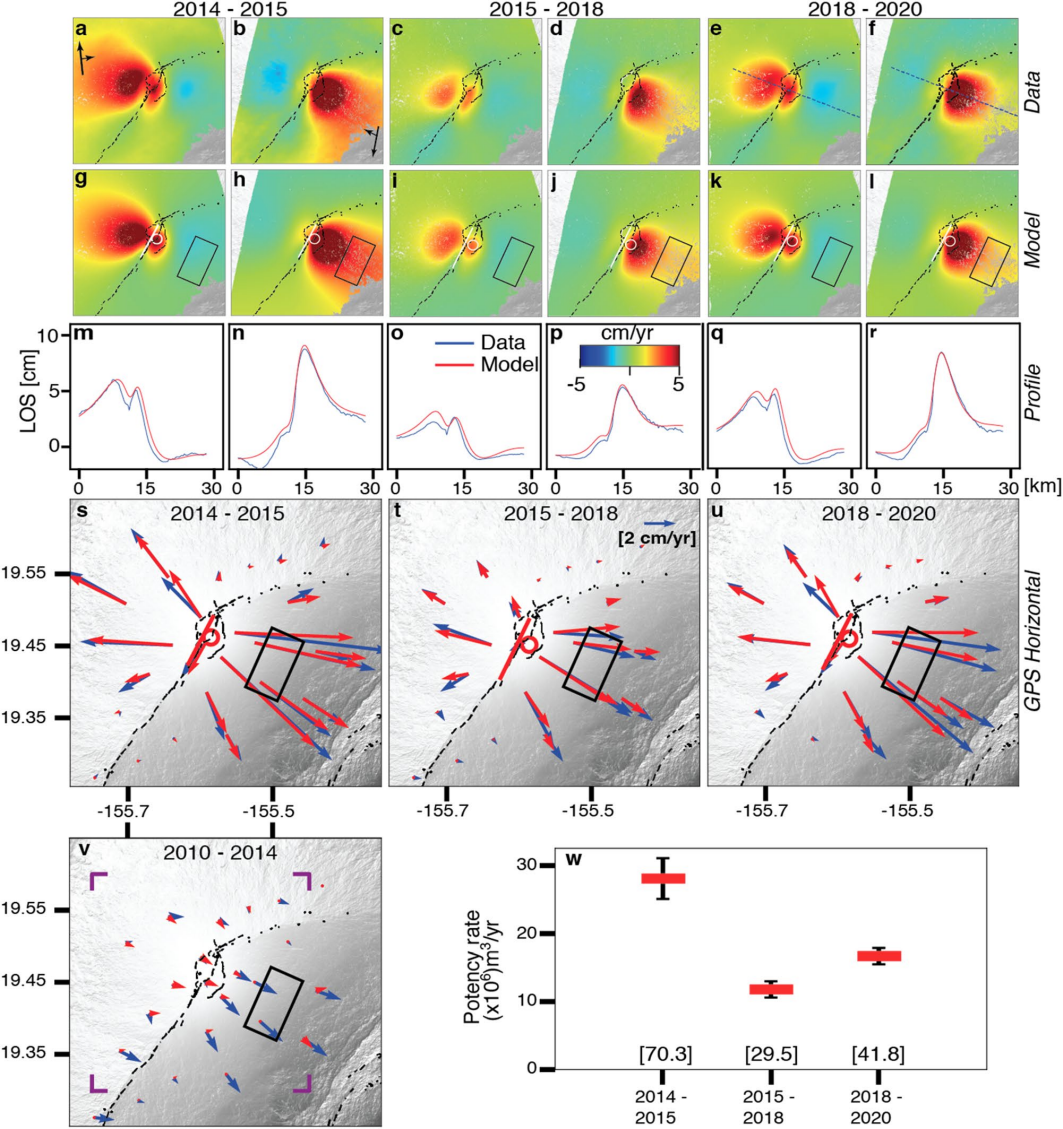
InSAR and GPS data together with modelling results for the three time periods. (**a**–**f**) Ascending and descending InSAR velocities, (**g**–**l**) best-fitting model predictions, (**m**–**r**) data and model predictions in a profile perpendicular to the rift zone, (**s**–**u**) GPS data (red) and model predictions (blue). (**v**) GPS data and model prediction for the 2010–2014 period. (**w**) Potency rates of the dike-like magma body. Bottom values in (**w**): total potency for the time period. White line: opening dislocation; White circle: mogi source; black rectangles: dislocation along decollement; blue dotted line: profile location for m-r; purple corners: Area shown in (**a**–**l**).

We assume a homogeneous elastic half space and model the deformation by an opening dislocation (assumed to be vertical), a mogi source and a rift-perpendicular 10 × 5 km^2^-sized shear dislocation under the eastern flank along the basal decollement at the paleo-seafloor, representing seaward motion along a sub–horizontal fault. We selected this model after testing a variety of models (see Supplementary Information S2). In order to estimate the total flux of magma into the shallow system, we combine the potency rates of the dislocation (surface area × opening rate) with the potency rates of the Mogi source (1.8 × volume change rate assuming a Poisson’s ratio of 0.25^16^). The InSAR and GPS data (see Supplementary Table S1.1) for the three time periods together with the model predictions are shown in Fig. 2a–u. The preferred models consist of dislocations with length × down-dip dimensions of 8 × 8.5, 10 × 3, and 9 × 4 km^2^, upper edge at 3.1, 2.5 and 2.5 km depth beneath the summit, and opening at rates of 0.3, 0.2 and 0.25 m/year, for the three periods, respectively, combined with Mogi sources at 4.4, 4.2 and 3.9 km depth below the summit with about 40–80% potency of the dislocations (see Supplementary Information S2). Both the 2015 decrease in vertical extent of the dike (from ~ 8.5 to ~ 3 km) and the upward migration of the upper dislocation edge (by ~ 0.6 km) are robust inversion results (Supplementary Fig. S1.13). Potency rates for the dike-like magma body are 28.1 ± 3, 11.8 ± 1 and 16.7 ± 1 m^3^/year for the three time periods, respectively (Fig. 2w). The decollement fault patch under the eastern flank is slipping at rates of 0.21, 0.22 and 0.27 m/year during the three periods, respectively.

For the 2010–2014 period the GPS velocities are well explained by decollement slip along the eastern flank with potency corresponding to a moment magnitude of M_w_6.0 (slip rate of 0.33 m/year along a 10 × 5 km^2^ patch, Fig. 2v).

We attribute differences between the observations and model predictions (Fig. 2m–u) to the assumption of homogeneous and isotropic material properties, the neglection of the elastic effects of the topography^17^, and to the simplified representation of the magma body by a combination of an opening dislocation and a Mogi source. Though we account for the elevation of the data points (see Supplementary Information S2), use of isotropic material properties without topographic layering might have underestimated the depths of the sources^18,19^.

For the stress analysis that follows, we consider an elastic 2-D tensile crack (mode I). The excess pressure increase rate during each time period is given by P_e_ = b × E/(2 × (1 − ν^2^) × L, where *b* is the maximum opening rate, *L* the half width, *E* Young’s modulus and ν Poisson’s ratio^19,20^. We use *E* = 40 GPa inferred from seismic tomography (see Supplementary Information S3.2) and *ν* = 0.25. For *L,* we use 4.25 km during 2014–2015, 1.5 km during 2015–2018 and 2 km during 2018–2020. For the maximum opening rate *b* we use equivalent dislocation widening rates inferred from the combined cumulative potency rates assuming 8 × 8.5, 10 × 3 and 9 × 4 km^2^ dislocations for 2014–2015 and 2015–2018, 2018–2020, respectively. As the excess pressure rates vary significantly between the three time periods because of varying dike dimensions, we also calculate equivalent widening rates and the corresponding excess pressure rates assuming a 9 × 5 km^2^ dislocation for all three time periods (see Supplementary Table S2.4). We refer to these models as the varying-crack size and uniform-crack size models, respectively. Using these assumptions, we find 2014–2020 cumulative equivalent widening of 2.55 and 2.45 m, achieved by cumulative pressure increases of 25 MPa and 21 MPa in the varying and uniform-crack-size models, respectively.

## Discussion

### Shallow magmatic system

We found that almost two thirds (~ 61%) of the newly arriving magma during 2014–2020 accumulated in a tabular, dike-like body within the rift zone and the remainder in a shallow chamber represented in our model by a Mogi source. At Mauna Loa, magma accumulation in a subvertical dike-like magma body is a consequence of the stress field exerted by the topography^21^. Within the rift zone, the maximum principal stresses are vertical, and the least principal stresses are horizontal and rift-perpendicular^22^, enabling the intrusion of magma into a dike-like body aligned with the rift zone. The Mogi source likely represents a geometric complexity of the magma body and not a stationary magma chamber as indicated by the southward shift of this source in 2015 which is a robust inversion result (see Supplementary Information S2.12). It could represent sill-type magma storage facilitated by shallow extensional unloading stress from the caldera collapse ~ 600 years ago^6,23^.

### Vertical stagnation of the dike-like magma body

Our analysis has shown that since 2002 the upper edge of the inflating dike-like magma body has remained below ~ 2.5 km beneath the summit. This suggests that the vertical ascent of the body was arrested by a stress barrier, most likely by layers with varying mechanical properties^24^. Studies have shown that the presence of a stiffer layer can hinder the ascent of a vertically propagating dike^25^ as can a more compliant layer^24,26^. In addition, negative buoyancy from magma entering into a layer with lower density than the magma can also hinder dike ascent^3,27^, although density layering appears to play a subordinate role for lateral and vertical dike propagation compared to rigidity layering^25^.

### Lateral growth following topographic stress gradient

In a lithostatic crust, a quasi-static, dike will grow and evolve maintaining a balance of equilibrium between the excess magma pressure and the ambient normal stress acting on the dike wall. While the excess pressure in the dike depends on the excess pressure in the source region, the flux into the dike, the magma compressibility and the viscosity^28^, the ambient normal stress is the result of perturbations to the lithostatic stress due to tectonic stress from plate motion, edifice loading, surface mass redistributions (e.g. caldera collapse, landslides) and residual stress from past events such as earthquakes, slow slip events and previous magma intrusions^7,29^. At Mauna Loa, which is not subject to tectonic stress, stress field perturbations arise from slip along the basal decollement, magma intrusions, and topographic load.

**Figure 3:**
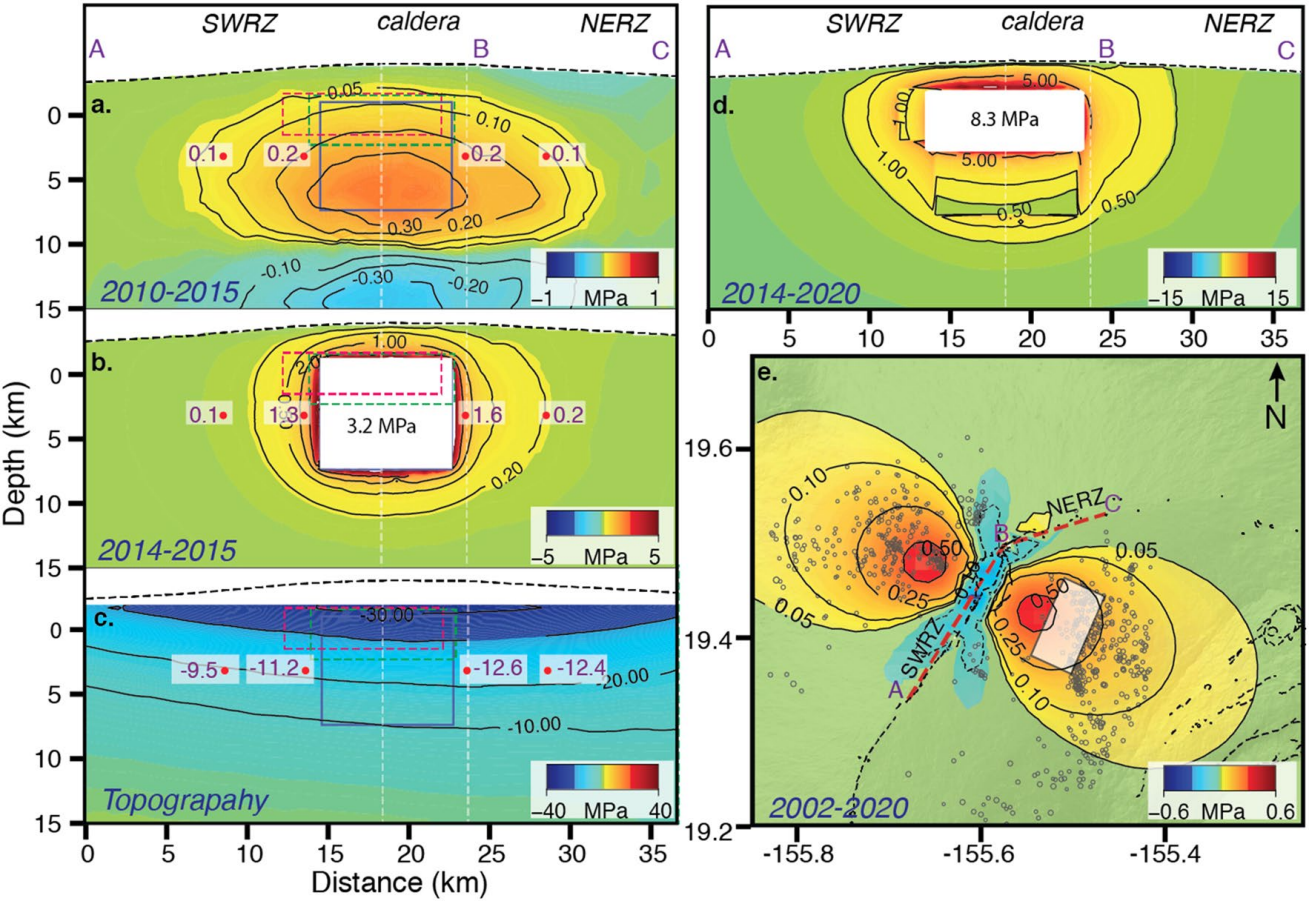
Perturbations in normal stress along Mauna Loa’s rift zone due to (**a**) 2010–2015 slip along decollement fault (1.6 m along a 10 × 5 km^2^ fault patch), (**b**) 2014–2015 excess pressure (3.2 MPa on 8 × 8.5 km^2^ dike-like magma body) and (**c**) the topography. (**d**) Cumulative normal stress due to 2014–2020 rift widening. (**e**) Shear stress perturbation along the decollement due to 2002–2020 inflation of the magma body (receiver fault strike is 207° and 27° east and west of the rift zone, respectively. In (**a**–**c**) Blue, Red, Green rectangles show modelled dislocation for 2014–2015, 2015–2018, 2018–2020 time periods respectively. Numbers in white shading indicate stress at that location.

Figure 3 shows the normal stress perturbations in direction perpendicular to the rift zone due to the 2010–2015 decollement slip, due to the 2014–2015 inflation of the dike-like magma body, and due to topographic loading, the latter simulated by applying a vertical compressional normal stress due to the topography above 1.7 km elevation to the surface of an elastic half space^4,6,30^(see Supplementary Information S3). Decollement slip imparted 0.2 MPa normal stress (tensional) in the area of the dike-like magma body (Fig. 3a), 80% of which was imparted prior to 2014, facilitating magma intrusion. Pressurization of the magma body imparted up to 3 MPa tensional stress symmetrically around its margins, rapidly declining with distance (Fig. 3b). The compressional stress field from the topographic load is asymmetric around the body (Fig. 3c). At 1 and 6 km from the southern and northern margins of the magma body the normal stress is − 9.5 and − 11.2 MPa, and − 12.6 and − 12.4 MPa, corresponding to along-rift gradients of 0.34 and 0.04 MPa/km, respectively. The stress gradient due to decollement slip is negligible (Fig. 3a), as are the stress concentrations at the margins of the body when treated as a cavity (see Supplementary Information S3.4). This suggests that the topographic stress gradient facilitated the southward growth of the dike-like magma body. The magnitude of the topographic stress may be smaller than modelled because the volcanic edifice was built over a long time during which stresses might be redistributed^29,31^.

### Stress perturbation along decollement fault and earthquake potential

To understand how magma intrusion affects the stability of the volcano, we also evaluate the stress changes along the decollement fault. The previous eruptions of Mauna Loa were preceded by M > 6 decollement earthquakes underscoring their role in perpetuating eruptions^12^. The 2002–2020 cumulative widening of the magma body by 4.7 m produced up to 0.6 MPa rift-perpendicular shear stress along the decollement fault (Fig. 3e). A finite element model predicts that for a 20 × 20 km^2^ fault under the eastern flank, the imparted seaward slip is 0.1 m, corresponding to a moment magnitude of M_w_5.9 (see Supplementary Table S3.5.1), which is less than the geodetically inferred slip corresponding to M_w_6.3 during this time period (see Supplementary Table S2.4). A possible explanation for the difference is that decollement slip is not only driven by dike widening but also by gravitational spreading of a central, above-decollement, ductile magma mush as suggested for Kilauea^32^. However, given that our data don’t constrain the location of decollement slip, we can’t completely rule out the possibility that portions of the fault such as the Kaoiki area near Kilauea maintain enough shear stress to generate a significant earthquake.

The shear stress imparted along the western decollement is of similar magnitude as on the eastern decollement (Fig. 3e) and corresponds to a M_w_5.9 earthquake for a 20 × 20 km^2^ slip patch and a M_w_6.3 for a 30 × 30 km^2^ patch. But this fault has not moved since 2002.

### Stress state around the magma body

The 2014–2020 intrusion resulted in > 15 MPa of tensional normal stress above the dike-like magma body and 5 and 10 MPa to south and north of it (Fig. 3d), respectively. This north–south difference in stress magnitude occurs because the 2015 southward growth relieved previously imparted stress in that area, and because the northern tip of the magma body did not inflate during 2015–2018. The 2015 upward shift relieved some of the previously imparted stress at the upper margin of the magma body. The magma pressure increase during this period was higher (more than 20 MPa in the uniform crack-size model and 21.6 MPa in the varying crack-size model) but the imparted tensional stress falls off rapidly with distance.

The inferred excess pressure during the entire 2002–2020 period of 37 MPa is significantly higher than the in-situ tensile strength of basaltic rock of 0.5–9 MPa^19,33^, which is sometimes considered as the failure pressure threshold for dike injection. Such high excess pressures can be partially supported by compressional stress from the topography. Furthermore, the inferred excess pressure could be an overestimate because the local Young’s modulus might be an order of magnitude smaller than the average Young’s modulus estimated from the seismic wave speeds which we used. Another potential bias is that the crack model used for excess pressure determination implicitly assumes a lithostatic stress field^2^. However, the pore fluid pressure distribution in basaltic volcanoes is likely hydrostatic, which also affects the pressure threshold for tensile failure of a magma chamber^34,35^.

### Evolution of the dike-like magma body

**Figure 4:**
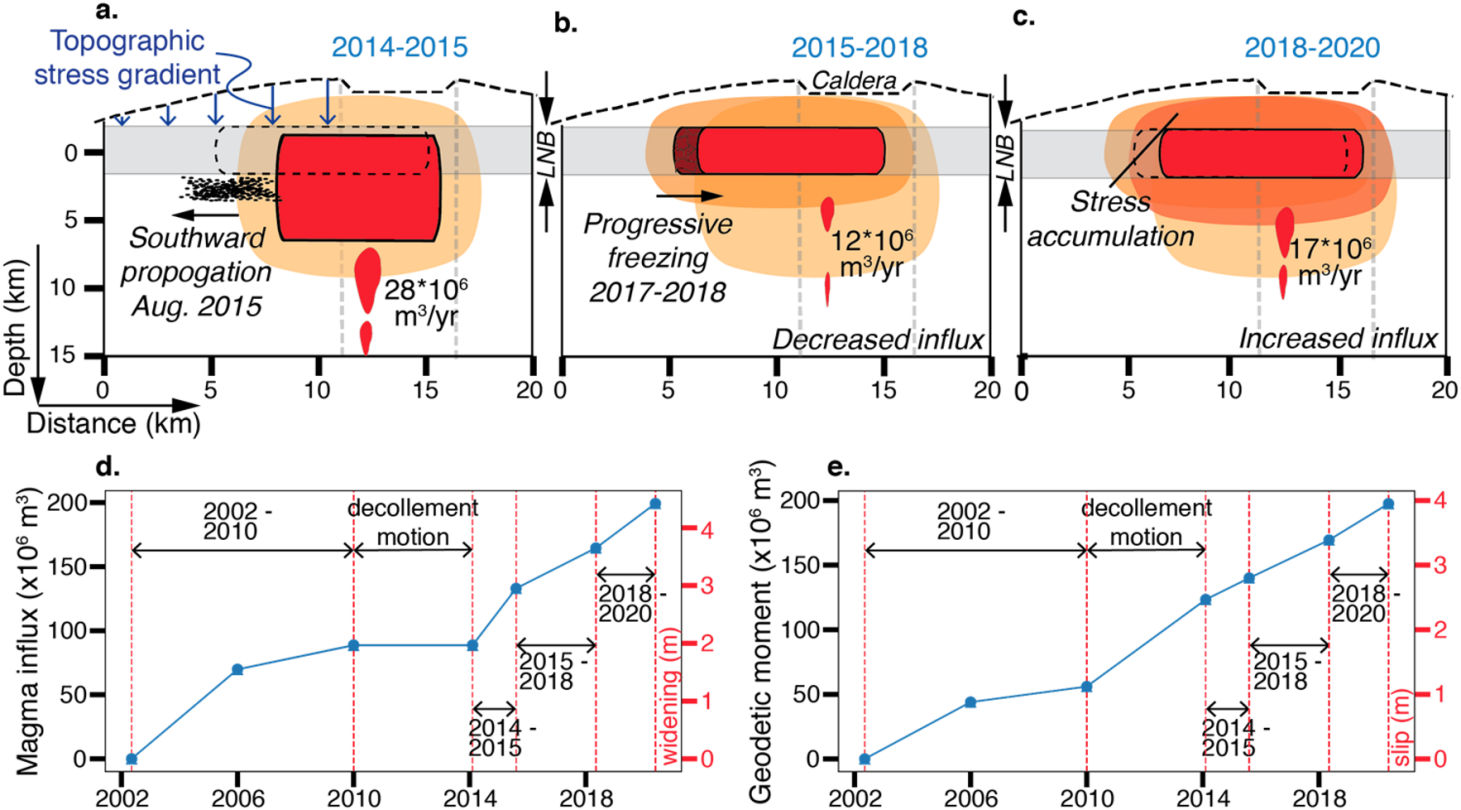
Schematic illustration of the evolution of Mauna Loa’s magma body during the three time periods. Blue arrows in (**a**): stress from topography; black dots: seismicity; black dotted line: position of dike-like magma body during 2015–2018 period; gray shadings: layer of neutral buoyancy (LNB, 2.5–5 km depth below the summit^39^); orange-color shadings: tensional stress (darker shadings: increasing stress magnitude). (**d**) Cumulative magma influx into the dike-like magma body since 2002 and corresponding widening on a 9 × 5 km^2^ dike-like magma body and (**e**) cumulative geodetic moment on the eastern decollement fault since 2002 and the corresponding slip on a 10 × 5 km^2^ fault (see Supplementary Table S2.4 for values). The dike-like magma body had the largest width (vertical extent) during 2014–2015. It is shown during 2018–2020 with the width of 2015–2018 because we don’t consider the inversion result of width increase to be significant.

The evolution of the magma body is summarized in Fig. 4. During 2014–2015 a new batch of magma reached the shallow system. It increased the excess pressure, but not enough to overcome the sum of the compressive stress from the topographic load, the tensile strength of the overlying rock and the (likely) negative buoyancy to propagate upwards. Unable to travel upwards, the magma followed the topographic stress gradient to propagate laterally into the SWRZ (Fig. 4a), relieving some of the previously imparted extensional stress. A March to September 2015 cluster of seismicity at the southern end of the magma body (Fig. 1a,d) suggests the slow breakage of a barrier^3,36^. The southward shift of the deformation source was not associated with ground subsidence over its previous northern edge, probably because of volume expansion of the compressed magma^37^ and volatile exsolution^38^. In 2017 the deformation source started to progressively migrate back to it’s pre-2015 location (Fig. 4b,c). We hypothesize that the magma in the dike tip gradually froze because not sufficient hot, fresh magma was supplied. Both the 2015 decrease in vertical extent of the dike-like magma body as well as the upward migration of the upper edge are consistent with a vertically ascending dike starting to propagate laterally when reaching a stiffer layer forming a stress barrier^25^. Lateral propagation is also favored at the level of neutral buoyancy (LNB)^3^, which ranges at Mauna Loa from 2.5 to 5 km beneath the summit^39^.

The inferred magma influx and decollement slip since 2002 is summarized in Fig. 4d,e (see Supplementary Table S2.4). The averaged 2014–2020 potency rate of ~ 18 × 10^6^ m^3^/year was ~ 90% of the 1875–1940 average eruption rate^40^, a period when the volcano had a high level of activity. The volume of accumulated magma since 2002 (0.2 km^3^) is nearing the volume of the lava flows of the 1984 eruption (0.22 km^3^)^41^. The geodetic moment of slip along the eastern decollement fault corresponds to 4 m along a 10 × 5 km^2^ fault (Fig. 4e).

### Potential for upward propagation

Will continuing magma influx and tensional stress increase overcome the stress barrier and lead to sudden upward injection of a dike and to an eruption? The location of the maximum 2014–2020 tensional stress perturbation along the upper margin of the body (Fig. 3d) favours but does not necessitate upward dike propagation. The dike-like magma body could also grow northwards or inject a dike where the imparted tensional stress is 10 MPa less. This is because of unknown pre-2002 stress perturbations and the magnitude of the compressional stress from topographic loading is poorly constrained because an unknown portion could have been relieved over time^29^. In addition, previous caldera collapses might have resulted in rigidity layering acting as a stress barrier to dike ascent^5,25^. Nevertheless, additional stress is required for the magma to ascend beyond LNB as it needs to overcome negative buoyancy and compression from topography.

A M > 6.0 decollement earthquake as occurred prior to previous eruptions would be a game changer. Both the addition of tensional stress to the rift zone and decompression-induced excess pressure increase by magma-internal processes could change the course of magma migration^12^.

## Summary and conclusions

A new period of magma intrusion into Mauna Loa’s dike-like magma body under the southern caldera area started in 2014 and widened the magma body by ~ 2.5 m as of 2020. The 2014–2015 and 2018–2020 centers of uplift coincide with the inferred centers of deformation prior to, and during, the 1984 eruption^42,43^. Rift zone inflation was accompanied by steady aseismic decollement slip under the eastern flank. The averaged 2014–2020 potency rate of ~ 18 × 10^6^ m^3^/year was ~ 90% of the 1875–1940 average eruption rate^40^, a period when the volcano had a high level of activity. In 2015 the magma body grew southwards in response to increased magma pressure ~ 2.3 km down rift following the topographic stress gradient. But when the magma influx rate waned, the southern tip solidified, and the inflation continued in the pre-2015 position as of the time of this writing. Assuming isotropic material properties and strength, the stress state within the volcano suggests two possible future scenarios. In the first scenario, continuing pressurization of the magma body leads to upward or northward injection of a dike and possibly propagation to the surface. In the second scenario an M_w_ > 6 earthquake occurs along the western decollement fault. The earthquake causes rift zone unclamping, the growth of tensile stress concentrations around the magma body, and decompression-induced magma-internal excess pressure increase, all favoring the upward propagation of a dike.

## Supplementary Information

Supplementary Information.

## Data Availability

Cosmo SkyMED SAR imagery is available at SSARA-Seamless SAR Archive at the portal (https://web-services.unavco.org/brokered/ssara/gui). GPS data used in the study is available from Nevada Geodetic Laboratory at the portal (http://geodesy.unr.edu/NGLStationPages/stations). All the information needed for evaluating the results are given in the Supplementary Information. Code to reproduce are made available via github page: (https://github.com/geodesymiami/2021_MaunaLoa_Varugu_Amelung) and necessary data is placed in zenodo (https://zenodo.org/record/4677624#.YHDPnhNKhTY) for easy access. Any other code or FEM model files will be provided up on reasonable request to the corresponding author.

## References

[1] Anderson EM (1936). Dynamics of formation of cone-sheets, ring-dikes, and cauldron subsidences. Proc. R. Soc. Edinb..

[2] Delaney, P. T., & Pollard, D. D. Deformation of host rocks and flow of magma during growth of minette dikes and breccia-bearing intrusions near Ship Rock, New Mexico. *US Geol. Surv. Prof. Pap.* **1202**, 1–61 (1981).

[3] Rubin AM (1995). Propagation of magma-filled cracks. Annu. Rev. Earth Planet. Sci..

[4] Pinel V, Jaupart C (2004). Magma storage and horizontal dyke injection beneath a volcanic edifice. Earth Planet. Sci. Lett..

[5] Corbi F, Rivalta E, Pinel V, Maccaferri F, Bagnardi M, Acocella V (2015). How caldera collapse shapes the shallow emplacement and transfer of magma in active volcanoes. Earth Planet. Sci. Lett..

[6] Xu W, Rivalta E, Li X (2017). Magmatic architecture within a rift segment: Articulate axial magma storage at Erta Ale volcano, Ethiopia. Earth Planet. Sci. Lett..

[7] Einarsson P, Brandsdóttir B (1980). Seismological evidence for lateral magma intrusion during the July 1978 deflation of the Krafla volcano in NE-Iceland. J. Geophys..

[8] Heimisson ER, Hooper A, Sigmundsson F (2015). Forecasting the path of a laterally propagating dike. J. Geophys. Res. Solid Earth.

[9] Walter TR, Amelung F (2004). Influence of volcanic activity at Mauna Loa, Hawaii, on earthquake occurrence in the Kaoiki Seismic Zone. Geophys. Res. Lett..

[10] Amelung F, Yun SH, Walter TR, Segall P, Kim SW (2007). Stress control of deep rift intrusion at Mauna Loa volcano, Hawaii. Science.

[11] Lockwood JP, Lipman PW (1987). Holocene eruptive history of Mauna Loa volcano. U.S. Geol. Surv. Prof. Pap..

[12] Walter TR, Amelung F (2006). Volcano–earthquake interaction at Mauna Loa volcano, Hawaii. J. Geophys. Res. Solid Earth.

[13] Miklius A (1995). Recent inflation and flank movement of Mauna Loa volcano. Geophys. Monogr..

[14] Okubo PG, Wolfe CJ (2008). Swarms of similar long-period earthquakes in the mantle beneath Mauna Loa Volcano. J. Volcanol. Geotherm. Res..

[15] Macdonald, G. A. & Hubbard, D.H. *Volcanoes of Hawaii National Park *Vol. 4, No. 2 (Naturalist Division, Hawaii National Park, 1951).

[16] Bonafede M, Ferrari C (2009). Analytical models of deformation and residual gravity changes due to a Mogi source in a viscoelastic medium. Tectonophysics.

[17] Cayol V, Cornet FH (1998). Effects of topography on the interpretation of the deformation field of prominent volcanoes—Application to Etna. Geophys. Res. Lett..

[18] Williams CA, Wadge G (1998). The effects of topography on magma chamber deformation models: Application to Mt. Etna and radar interferometry. Geophys. Res. Lett..

[19] Gudmundsson A (2020). Volcanotectonics: Understanding the Structure, Deformation and Dynamics of Volcanoes.

[20] Hamling IJ, Kilgour G (2020). Goldilocks conditions required for earthquakes to trigger basaltic eruptions: Evidence from the 2015 Ambrym eruption. Sci. Adv..

[21] Fiske RS, Jackson ED (1972). Orientation and growth of Hawaiian volcanic rifts: The effect of regional structure and gravitational stresses. Proc. R. Soc. Lond..

[22] Dieterich JH (1988). Growth and persistence of Hawaiian volcanic rift zones. J. Geophys. Res. Solid Earth.

[23] Lipman PW (1995). Declining growth of Mauna Loa during the last 100,000 years: Rates of lava accumulation vs. gravitational subsidence. Geophys. Monogr..

[24] Gudmundsson A, Brenner SL (2004). Local stresses, dyke arrest and surface deformation in volcanic edifices and rift zones. Ann. Geophys..

[25] Urbani S, Acocella V, Rivalta E (2018). What drives the lateral versus vertical propagation of dikes? Insights from analogue models. J. Geophys. Res. Solid Earth.

[26] Gudmundsson A (2002). Emplacement and arrest of sheets and dykes in central volcanoes. J. Volcanol. Geotherm. Res..

[27] Lister JR, Kerr RC (1991). Fluid-mechanical models of crack propagation and their application to magma transport in dykes. J. Geophys. Res. Solid Earth.

[28] Buck WR, Einarsson P, Brandsdóttir B (2006). Tectonic stress and magma chamber size as controls on dike propagation: Constraints from the 1975–1984 Krafla rifting episode. J. Geophys. Res. Solid Earth.

[29] Rivalta E (2019). Stress inversions to forecast magma pathways and eruptive vent location. Sci. Adv..

[30] Pinel V, Jaupart C (2000). The effect of edifice load on magma ascent beneath a volcano. Philos. Trans. R. Soc. Lond. A..

[31] Maccaferri F, Richter N, Walter TR (2017). The effect of giant lateral collapses on magma pathways and the location of volcanism. Nat. Commun..

[32] Plattner C, Amelung F, Baker S, Govers R, Poland M (2013). The role of viscous magma mush spreading in volcanic flank motion at Kīlauea Volcano, Hawai ‘i. J. Geophys. Res. Solid Earth.

[33] Acocella V (2021). Volcano-Tectonic Processes.

[34] Gerbault M (2012). Pressure conditions for shear and tensile failure around a circular magma chamber; insight from elasto-plastic modelling. Geol. Soc. Lond. Spec. Publ..

[35] Albino F, Amelung F, Gregg P (2018). The role of pore fluid pressure on the failure of magma reservoirs: Insights from Indonesian and Aleutian arc volcanoes. J. Geophys. Res. Solid Earth.

[36] Biggs J, Amelung F, Gourmelen N, Dixon TH, Kim SW (2009). InSAR observations of 2007 Tanzania rifting episode reveal mixed fault and dyke extension in an immature continental rift. Geophys. J. Int..

[37] Rivalta E, Segall P (2008). Magma compressibility and the missing source for some dike intrusions. Geophys. Res. Lett..

[38] Geshi N, Browning J, Kusumoto E (2020). Magmatic overpressures, volatile exsolution and potential explosivity of fissure eruptions inferred via dike aspect ratios. Sci. Rep..

[39] Ryan MP (1987). Neutral buoyancy and the mechanical evolution of magmatic systems. Magn. Process Physicochem. Princ. Geochem. Soc. Spec. Publ..

[40] King CY (1989). Volume predictability of historical eruptions at Kilauea and Mauna Loa volcanoes. J. Volcanol. Geotherm. Res..

[41] Barnard WM (1995). Mauna Loa Volcano: Historical eruptions, exploration, and observations (1779–1910). Geophys. Monogr..

[42] Decker RW (1983). Seismicity and surface deformation of Mauna Loa volcano, Hawaii. EOS Trans. Am. Geophys. Union.

[43] Lockwood JP (1985). The 1984 eruption of Mauna Loa Volcano, Hawaii. EOS Trans. Am. Geophys. Union.

