# Supplementary material for: Southward growth of Mauna Loa’s dike-like magma body driven by topographic stress
Source: Sci Rep. 2021 May 10;11:9816. doi: 10.1038/s41598-021-89203-6 (PMC8110994; doi:10.1038/s41598-021-89203-6)
Supplement: Supplementary file 1 — Supplementary Information. [file 41598_2021_89203_MOESM1_ESM.pdf]

Supplemental Material for

Southward growth of Mauna Loa's dike-like magma body driven by topographic stress

Bhuvan Varugu[1*], Falk Amelung[1]

[1]Rosenstiel School of Marine and Atmospheric Sciences, University of Miami, 4600 Rickenbacker Causeway, Miami, FL 33149, USA.

*Correspondence to: bvarugu@rsmas.miami.edu

This file contains:

Data and Methodology in sections S1-S3.

Figs. S1.1 to S3.6

Tables S1.1 to S3.5.2

S1: InSAR and GPS data

InSAR

We use 136 ascending (track 91) and 142 descending pass (track 10) Cosmo Skymed stripmap images with a spatial resolution of 3 m and median temporal spacing of 16 days spanning June 2013 to May 2020. We use the ISCE software interferograms generation and the Miami INsar Time-series software in PYthon (MintPy) software[44] for time-series processing using the small baseline (SB) approach. We used all interferograms with spatial baseline of less than 900 m and temporal separation of less than 90 days, removed the topographic phase contributions using a 10 m resolution DEM and applied 4 looks in both azimuth and range directions. For time-series analysis we use the MintPy routine processing workflow (no network modification, no unwrap error correction, weighted least squares inversion of the phase measurements using the inverse of the phase variance as weights, correction for topographic residuals using the approach in[45], phase contributions from tropospheric delays removed using phase delay-elevation ratio[46]. The time-series are referenced to dates with least atmospheric contributions (01/10/2014 for the ascending and 03/09/2014 for the descending track). The average velocities are obtained by fitting a linear trend to the time-series. Finally, the InSAR data is geo-referenced with 30 m posting.

GPS

We use daily position time-series from 23 GPS stations covering Mauna Loa (the GPS stations without data gaps), processed by the University of Nevada Reno[47] starting in 2000 (IGS14 reference frame). The daily solutions were formed into a time series and average velocities are obtained by fitting a linear polynomial to the data using inverse of the data variance as weights. The resulting velocities are then referenced to the GPS station on Mauna Kea (MKEA) (see Table S1.1 and Fig. S1.2).

Description of data

The pseudo-position plot (Fig. S1.3) shows how summit deformation evolved since 2010. The stations on the eastern flank of the volcano moved steadily until early 2014 when they started to accelerate (stations PAT3, ALAL). In contrast, the stations on the western flank (PHAN, SLPC) were stable but started to move in mid 2014. Several stations located around the summit caldera show a change in direction in August 2015 and again in April-May 2018. The timing of the 2018 change is difficult to discern in the daily position plot because of the co-seismic displacements due to the May 4 Kilauea earthquake.

Fig. S1.4 addresses the question whether the 2017-2018 shift of the inflation source was instantaneous or occurred over a longer period. Figs 1.4e,f shows that during January 2017 to April 2018 the displacement pattern, although the data are noisy, was located a few hundred meters further north than during August 2015-January 2017 (Figs. S1.4c,d). This shows that there was a progressive

northward migration of the inflation source rather than an instantaneous shift, consistent with the gradual solidification of magma in the tip of the dike.

Figs. S1.5 addresses the question whether the May 4 2018 Kilauea earthquake impacted the displacement pattern. The May 2018-May 2020 velocity (without the earthquake, Figs. S1.5i,j) is identical to the April 2018-May 2020 (Fig. S1.4g,h), indicating that the influence of the earthquake is negligible.

Summary of figures

Fig. S1.1 shows the Line-of-sight (LOS) velocity maps for ascending and descending passes and the LOS displacement time series for representative points. Fig. S1.2 shows the horizontal displacement time series of GPS stations since 2000. Fig S1.3 shows a horizontal pseudo-position plot since 2010. Fig. S1.4 shows the ascending and descending line-of-sight (LOS) velocities for the three time periods as discussed in the main paper, but period 2 is split into two shorter periods. Fig. S1.5 shows the LOS velocity for May 2018 (starting after the May 4 M_w6.9 Kilauea earthquake) to May 2020.

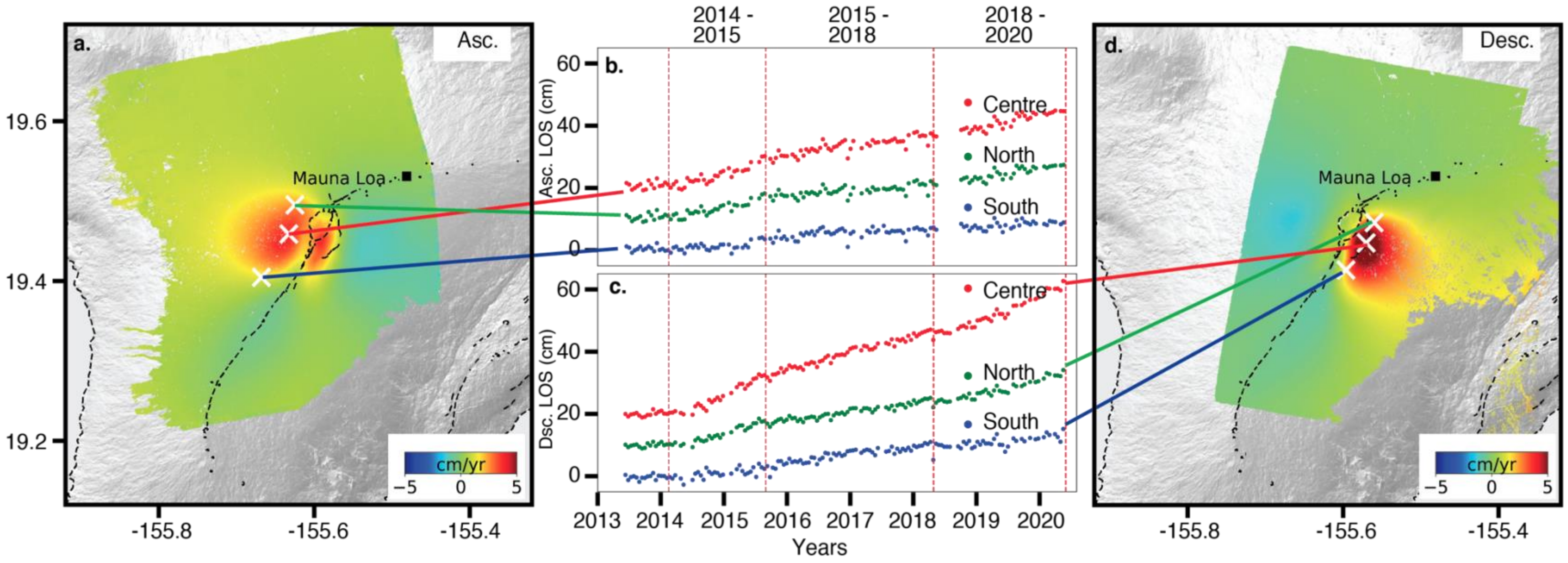

Figure S1.1. *Line-of-sight velocity maps for 2013-2020 from (a) ascending, (d) descending satellite passes (tracks 10 and 91, respectively), along with (b,c) displacement time series for three selected points near the central, northern and southern parts deforming area.*

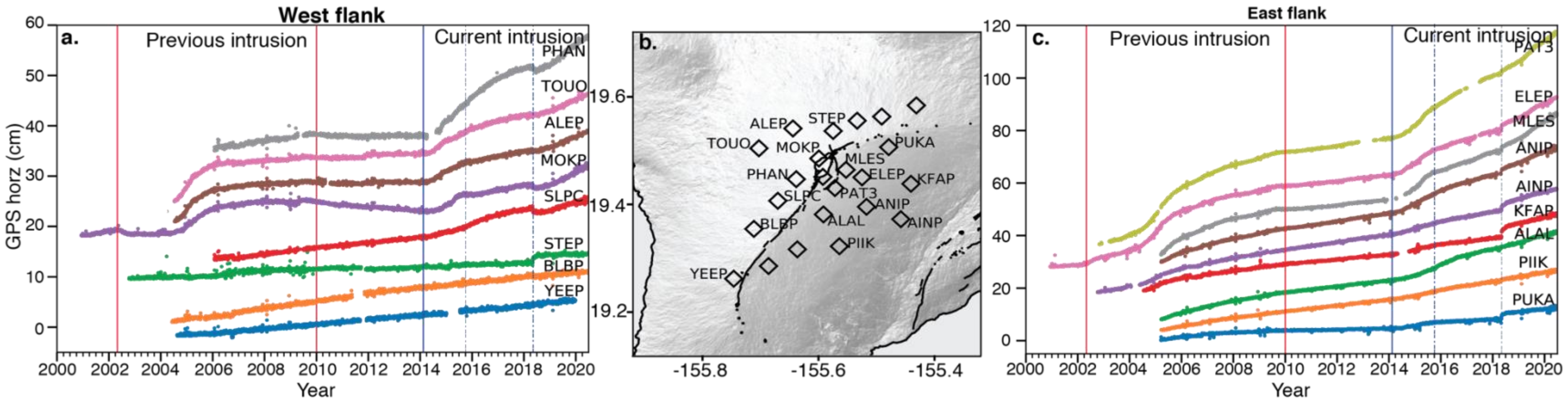

Figure S1.2 *2000-2020 GPS horizontal displacement for (a) western flank and (c) eastern flank of Mauna Loa. (b) GPS station locations.*

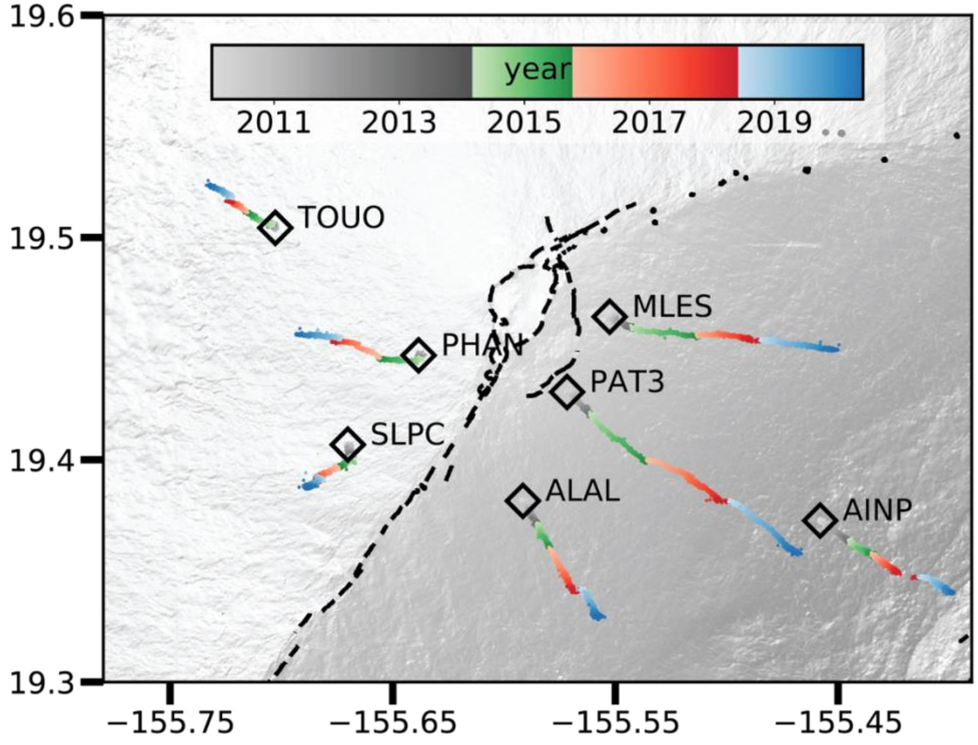

Figure S1.3. *Pseudo-position plot (displacement scaled to position) for the selected GPS stations of Fig. 1b in main paper.*

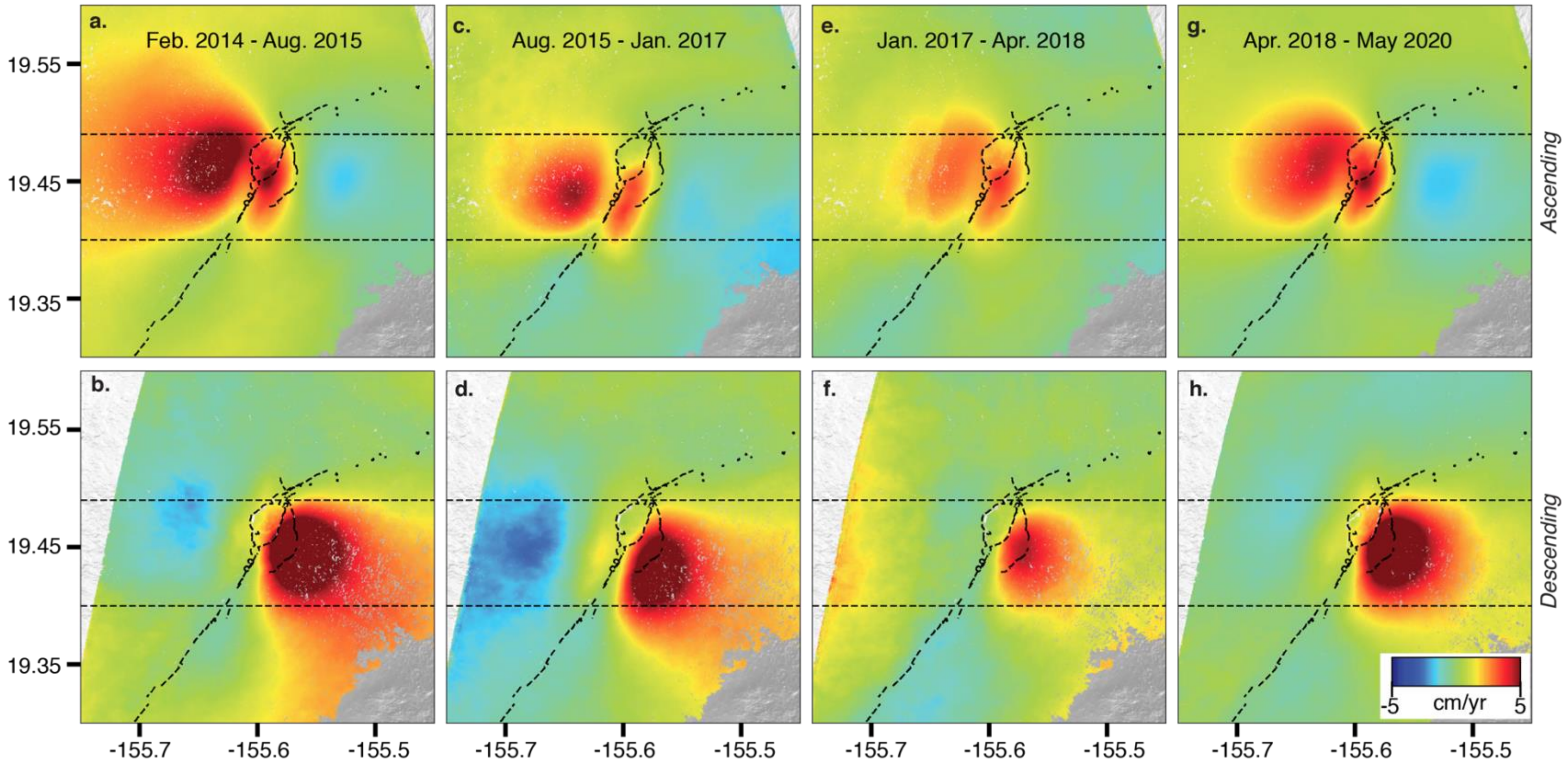

Figure S1.4. *Ascending and descending velocities for time periods (a,d) February 2014 - August 2015, (b, e) August 2015-Jan 2017, (c, f) January 2017-April 2018, (g,h) April 2018 - May 2020. Black dotted lines are to highlight the shift of deformation during 2015-2018.*

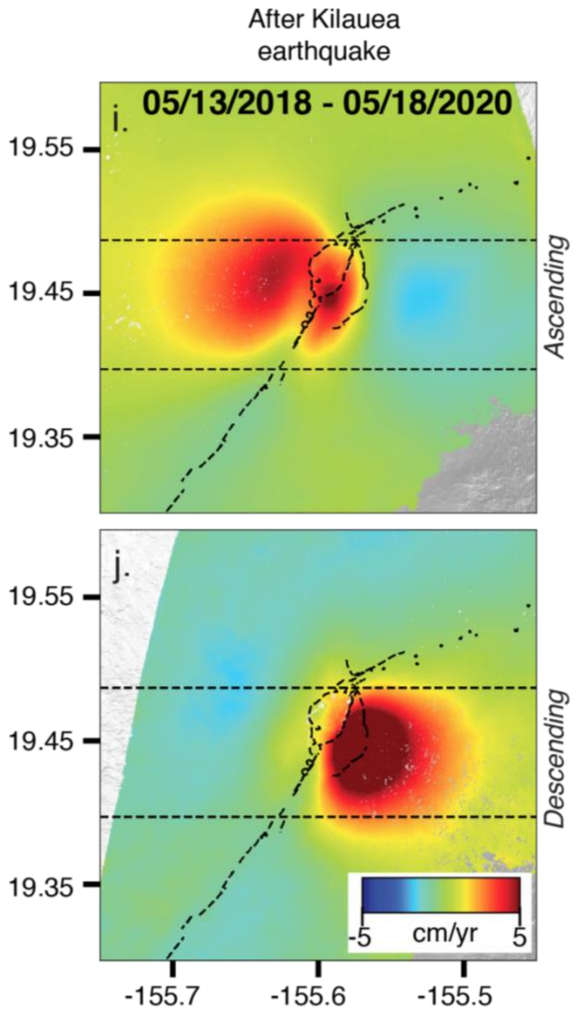

Figure S1.5. *Similar to Fig. 1.4 g, h but starting May 13 2018 after the May 4 2018 M_w6.9 earthquake at Kilauea.*

Table S1.1 *GPS velocities for the three time periods used in the modelling*

**Site**	**Lat**	**Lon**	**Feb. 2014 – Aug. 2015 (Time period 1)**						**Aug. 2015 – Apr. 2018 (Time period 2)**						**Apr. 2018 – May. 2020 (Time period 3)**					
			Nvel	Nerr	Evel	Eerr	Uvel	Uerr	Nvel	Nerr	Evel	Eerr	Uvel	Uerr	Nvel	Nerr	Evel	Eerr	Uvel	Uerr
**AINP**	-155.458	19.373	-13.84	0.91	23.07	1.20	-4.87	6.91	-10.91	1.07	15.00	1.23	1.18	7.24	-14.49	1.40	26.12	2.26	-0.44	8.35
**ALEP**	-155.644	19.541	22.55	0.89	-14.88	1.11	1.52	5.91	8.50	1.06	-5.21	1.16	2.77	6.59	15.95	1.36	-10.88	1.64	0.22	7.66
**BLBP**	-155.711	19.355	-3.92	0.83	3.25	1.08	-6.43	5.91	-4.68	1.04	2.98	1.17	-0.89	6.69	-3.72	1.22	3.63	1.49	-3.05	7.81
**ELEP**	-155.525	19.450	-14.51	0.85	61.65	1.58	11.09	6.05	-7.26	1.15	29.90	1.43	7.39	6.54	-14.06	1.30	50.63	1.79	10.07	7.47
**KFAP**	-155.441	19.438	-5.16	0.92	21.25	1.27	-5.42	6.52	-4.39	1.10	10.67	1.20	1.37	7.07	-8.60	1.72	20.58	2.71	-1.38	8.54
**MLCC**	-155.491	19.563	2.73	0.87	4.59	1.16	-5.26	5.98	-0.97	1.09	-0.43	1.13	0.29	6.69	-0.45	1.46	1.49	1.68	-2.36	7.68
**MLSP**	-155.592	19.451	-18.52	1.21	-8.78	1.22	45.92	7.40	-4.34	1.40	-7.30	1.18	34.26	6.52	-16.01	1.44	-11.31	2.42	52.77	8.36
**MOKP**	-155.599	19.485	18.57	1.26	-15.83	1.59	28.54	6.73	7.60	1.05	-5.13	1.24	14.33	6.59	16.81	1.60	-17.21	2.51	27.12	8.23
**PAT3**	-155.572	19.430	-51.42	1.51	57.19	2.06	39.21	7.53	-27.48	1.19	44.90	2.19	37.17	7.52	-46.96	1.64	55.46	2.33	42.52	8.07
**STEP**	-155.575	19.536	3.74	0.85	3.39	1.05	-2.65	5.97	0.11	1.08	1.65	1.12	1.07	6.71	2.17	1.37	2.94	1.61	-0.37	7.59
**TOUO**	-155.703	19.504	14.74	0.84	-25.08	1.20	-0.64	5.71	7.31	1.17	-11.20	1.20	2.55	6.52	10.22	1.29	-17.84	1.63	0.59	7.71
**YEEP**	-155.746	19.262	0.14	0.83	5.51	1.12	-6.93	6.09	-0.50	1.03	3.90	1.27	-0.26	6.98	1.15	1.21	4.78	1.45	-2.36	7.77
**ALAL**	-155.592	19.381	-24.59	1.08	11.97	1.13	2.78	6.12	-26.40	1.56	13.81	1.42	9.89	7.00	-24.04	1.43	13.52	2.23	5.92	8.36
**ANIP**	-155.517	19.396	-27.63	1.01	37.72	1.48	0.62	6.33	-17.03	1.06	25.18	1.54	6.51	6.78	-23.04	1.44	31.89	2.68	4.65	9.07

[Contd ….]

[Contd ….]

Site	Lat	Lon	Feb. 2014 – Aug. 2015 (Time period 1)						Aug. 2015 – Apr. 2018 (Time period 2)						Apr. 2018 – May. 2020 (Time period 3)					
			Nvel	Nerr	Evel	Eerr	Uvel	Uerr	Nvel	Nerr	Evel	Eerr	Uvel	Uerr	Nvel	Nerr	Evel	Eerr	Uvel	Uerr
**KHKU**	-155.637	19.317	-2.19	0.86	3.53	1.07	-9.51	5.92	-2.94	1.07	2.18	1.15	-2.69	6.56	-1.36	1.33	3.59	2.11	-3.86	8.03
**KNNE**	-155.686	19.286	-0.44	0.79	4.83	1.05	-6.78	5.63	-0.82	1.03	3.35	1.14	-2.69	6.41	1.39	1.31	4.36	2.03	-5.58	7.97
**MLES**	-155.553	19.464	-9.06	0.86	79.48	1.58	38.04	6.05	-2.41	1.26	34.19	1.25	18.81	6.67	-8.60	1.33	62.21	2.28	31.87	7.91
**MLRD**	-155.533	19.556	0.48	0.89	3.81	1.06	-5.12	6.08	-2.15	1.09	0.56	1.13	0.48	6.74	-1.18	1.56	1.89	2.38	-1.35	8.64
**PHAN**	-155.638	19.447	0.74	0.82	-41.47	1.92	25.30	6.07	10.26	1.77	-28.19	1.92	22.88	7.58	3.56	1.26	-34.50	2.04	21.29	7.72
**PIIK**	-155.564	19.322	-14.00	0.89	10.94	1.13	-4.28	5.96	-13.15	1.07	8.64	1.16	4.26	6.48	-12.36	1.35	10.95	2.08	1.52	7.97
**PUKA**	-155.479	19.506	5.58	0.83	15.85	1.33	-2.43	6.41	0.34	1.09	4.95	1.20	1.06	6.85	1.87	1.58	11.46	2.32	-0.74	8.34
**RADF**	-155.431	19.584	3.59	0.84	4.18	1.09	-7.82	6.28	0.45	1.09	-0.59	1.13	0.06	6.74	0.83	1.64	1.05	1.67	-1.08	7.63
**SLPC**	-155.670	19.407	-10.21	0.83	-11.88	1.28	-0.69	6.06	-8.62	1.04	-13.52	1.70	5.37	6.88	-8.43	1.38	-10.70	2.41	1.61	8.72

S2: Magma source modelling

Modelling approach

We model the magma sources as rectangular, uniform opening dislocations[48] and point sources[49], and the decollement fault underneath the volcanic pile as a shear dislocation, all in an isotropic elastic half space (Poisson's ratio of 0.25). We use a Bayesian inversion method to determine the best fitting source models which minimize the residual between the observed data and model predictions. We sample the LOS velocity maps and reduce the number of data points using an adaptive quadtree approach[50]. For each dataset we compute a variance-covariance matrix using an experimental semi-variogram[51] which is used to weight the data points using the inverse of their variance. We only use the horizontal GPS velocities owing to high noise levels in the vertical. We use the Geodetic Bayesian Inversion Software (GBIS)[52], which uses a Markov Chain Monte Carlo approach with Metropolis-Hastings algorithm. We account for the elevation effect of the topography using reference elevations obtained by comparing the displacement fields from a half-space model with those from computationally expensive varying-source depth models[18]; the reference elevations for each time period are given in Table S2.3).

To fit the data, we consider three sources: (i) inflation of a dike-like magma body in the rift zone, (ii) inflation of a magma chamber under the caldera, and (iii) slip along the basal decollement, represented by a uniform opening dislocation (referred to as dike), a Mogi source, and a uniform, slightly upward dipping dislocation, respectively, all embedded in a homogeneous elastic halfspace. We also tested the Compound Dislocation Model (CDM)[53], but obtained poorer fits. We consider various model configurations consisting of one or multiple sources and invert for the location, geometry and intensity (opening displacement, volume change and dip slip) with some constraints. For the dike we assume that it is vertical (dip fixed at 89.5°). For the decollement slip we assume uniform slip in direction perpendicular to the dike. As the data don't have the resolution to constrain the fault's location and size, we assume a 10*5 km^2-sized patch, constrained to be located along the basal decollement fault (at a depth of 10 km b.s.l. under the caldera, 5° upward dipping towards east, parallel to the dike). The best-fitting models are characterized by a minimum in Root Mean Square Error (RMSE) between data and model predictions, calculated as:

$$RMSE = \sqrt{\frac{([d-m]^2)_{InSAR} + w * ([d-m]^2)_{GPS}}{N}}$$

where d is data, m is model displacement and w is the weight of GPS data to InSAR data (1.0 in our case) and N is the total number of observations. Assuming suitable uniform priors (Table S2.2), we used a Markov Chain Monte Carlo based Bayesian inversion algorithm GBIS to derive posterior probability density functions (PDFs) for the model parameters. From the PDFs, we derive the optimal, mean

and variance ($2\boldsymbol{\sigma}$) for each model parameter. We estimated the model parameters using 1 million iterations and discarded the first 50000 iterations as initial burn-in[52,54].

Modelling results

We consider five different time periods, the three time periods discussed in the paper for which we have InSAR and GPS data (January 2014 to August 2015, August 2015 to April 2018, April 2018 to May 2020, referred to as time periods 1, 2 and 3), the prior time period for which we only have GPS data (January 2010 - February 2014 or 2010-2014) and an earlier time period studied by Amelung et al (2007)[10] for which we only use InSAR data (May 2002- December 2005, time period A).

For time periods 1, 2, 3 we considered three model configurations: a model consisting of a dike only (model A), a model consisting of a dike and a Mogi source (model B), and a model consisting of a dike, Mogi source and decollement fault (model C). The model configurations have 7, 11 and 12 free model parameters, respectively. For time period B for which the data have low signal-to-noise ratio, we consider a model of a decollement fault (model D) with only one free parameter (slip). For all time periods, the location of decollement is constrained by the model for the February 2014 to May 2018 period (combined time periods 1 and 2).

For each time period, we sub-sampled the InSAR data to around ~400 samples for each dataset. For the semi-variogram[51] used to construct the variance covariance matrix, we used a sill and nugget of $1\text{x}10^{-6}$ m^2 and $1\text{x}10^{-8}$ m^2 respectively. All the sources are allowed to vary within a set of non-informative priors (see Table S2.2).

The model fits for time periods 1,2,3 and are shown in Figs. S2.1 - S2.3. The model fit for time period B is shown in Fig. 2v in the main paper. The best-fitting model parameters along with variance (95 % certainty) are summarized in Table S2.1. The marginal and joint probability density distributions for models B and C for time periods 1,2 and 3 are shown in Figs S2.6 to S2.11. For comparison between different configurations, we also report the potencies for dislocation (surface area*opening), decollement fault (surface area * slip)[55] and mogi source (1.8*volume change)[16].

For the three periods, we can discard Model A because it is associated with LOS increase (subsidence) over the dike which is not observed (Fig. S2.1 - S2.3). The addition of a mogi source (Model B) leads to a better fit to the data. Our preferred configuration is model C which includes decollement slip. The dike is located under the summit caldera parallel to the rift and the mogi source is located under the eastern caldera rim. The potency of mogi source (see caption to table S2.1) is 40-80 % of the dike. Although models B and C have similar RMSE, we select model C because the addition of the decollement slip explains the GPS-observed east flank motion and is consistent with the elevated seismicity along the decollement (Fig. 1e).

The marginal and joint probability density distributions for the model configurations (models B and C) show that the model parameters are well constrained (Fig. S2.6-2.11) and in both configurations there is a negative correlation between dike width to dike depth and dike opening, as well as a positive correlation between dike opening and dike depth. Furthermore, for all three time periods model configuration C shows a positive correlation between the volume change of the mogi source and fault slip. 1D histograms of the location of the Mogi source and the dike vertical extent elucidate the changes between time periods (Fig. S2.12 and S2.13).

To compare our results with the previous intrusion (2002-2005, time period A), we modelled the data from Amelung et al.[10]. The model fit is presented in Fig. S2.4.

The geodetic data don't have the resolution to resolve the precise location of slip and/or temporal variations as the net velocities at the surface are less than 1 cm (see Fig. S2.5). The same surface displacements are produced for example by 1.5 meters slip along a 10*5 km^2 patch (as modelled) or by 0.25 m slip along a 30*10 km^2 patch (for more see table S3.5.1).

Reference elevation for the model depths

As we use elastic half space models, we have to account for the elevation of the data points which varies considerably. Williams and Wadge (1998)[18] do this by (i) adjusting for each data point the source depth based on a point's elevation and (ii) using the mean elevation of all data points as the reference elevation. This method is referred to as the depth-adjusted approach. Inversion using this method is computationally expensive as the Green's functions need to be calculated at every point at every iteration. We found that the obtained model parameters using this approach and the depth-not-adjusted approach are nearly identical, except for model parameters describing the source depths. We therefore used the depth-not-adjusted approach for model configuration A to estimate a reference elevation such that depth a.s.l. from both, the depth-not-adjusted and the depth-adjusted inversions are the same. This reference elevation is then used for all model configurations. We found for the three time periods reference elevations of 3325, 3939, and 3894 m above sea level respectively (Table S2.3).

Summary of source strengths

The strengths of the sources are described by the opening rate, slip rate, the lengths and widths of the dislocations, and the volume change rates of the Mogi source (Table S2.1). The corresponding potency rates for the five time periods are given in table S2.5. In order to obtain excess pressure rates on the complex magma body, we convert the potency rates into equivalent opening rates over the modelled dislocation, which then can be used to calculate the excess pressure rates (Table S2.4) (see section S3.1 for definitions). As this excess pressure is dependent on the size of the dislocation which varies over the three time periods, we also calculated excess pressure over an 9x5 km^2 dislocation. The two different configurations are referred to in the table as varying-crack size and uniform-crack size model, respectively.

Compound Dislocation Models.

Our best-fitting homogeneous elastic models for the magma system consist of closely spaced opening dislocation and a Mogi source. So, we also tried the Compound Dislocation Model (CDM[53]), which has higher degrees of freedom and would explain both planar intrusions and volumetric inflations. The CDM based models failed to converge toward a best fitting solution. The RMSE of the CDMs is 7 cm which is much higher than our preferred model.

Table S2.1 *Best-fitting model parameters for the various models for the three time periods. All uncertainties (±) are 2σ variations from the reported mean value. The depths are given with respect to sea level, the half space depths for the 9 models from top to bottom are {[(2.5)^{A},(2.2,3.5)^{B},(2.3,3.7,13)^{C}],[(2.9)^{A},(2.3,3.6)^{B},(2.3,4.1,13)^{C}],[(2.9)^{A},(2.3,3.4)^{B},(2.3,3.7,13)^{C}]}*

Model	Source	Length (km)	Width (km)	Depth^{+} (km) asl	Strike (°)	Dip* (°)	Easting^{++} (km)	Northing++ (km)	Opening rate /slip rate (m/yr)	Vol. change rate (Δv/yr)	Potency rate^ (x10^{6} m3/yr)	RMSE (mm/yr)	Figs.
**January 2014 - August 2015 (Time period 1)**													
A	Dike	7.1±0.2	2.7±0.6	0.8±0.1	22±0.6	89.5*	-0.27±0.03	0.5±0.07	0.52±0.08		9.9±0.6	9.2	S2.1; S2.6;S2.9
B	Dike	7.9±0.3	10.0±0.5	1.1±0.05	26±0.5	89.5*	0.05±0.05	0.5±0.07	0.27±0.01		22.±1.1	4.9	
	Mogi			-0.2±0.07			1.5±0.05	0.9±0.04		4.0±0.2	7.2±0.4		
C	Dike	8.1±0.03	8.6±1.2	1.0± 0.06	26±0.5	89.5*	0.04 ±0.04	0.4±0.08	0.28±0.02		20.2±2.5^{X}	4.9	
	Mogi			-0.3±0.06			1.5±0.05	0.9±0.05		4.5±0.2	8.1±0.4^{X}		
	Decollement	10 km*	5 km*	10 km*	204*	5*	8.3*	-2.7*$^{+}$	0.21±0.02		10.5±1.3^{X}		
**August 2015-April 2018 (Time period 2)**													
A	Dike	9.3±0.3	0.5±0.3	1.0±0.2	23±0.6	89.5	-0.7±0.05	-0.7±0.1	1.2±0.5		5.6±0.2	5.0	S2.2;S2.7;S2.10
B	Dike	9.6±0.2	3.0±0.7	1.6±0.2	24±0.6	89.5	-0.6±0.05	-0.9±0.1	0.22±0.06		6.4±0.6	3.9	
	Mogi			0.3±0.16			1.4±0.07	-1.7±0.1		2.1±0.2	3.8±0.4		
C	Dike	9.8±0.4	3.2±0.9	1.6±0.14	25±0.7	89.5	-0.6±0.06	-0.95±0.1	0.21±0.03		6.4±0.7^{X}	3.2	
	Mogi			-0.1±0.1			1.5±0.02	-0.3±0.1		3.0±0.2	5.4±0.4^{X}		
	Decollement	10 km*	5 km*	10 km*	204*	5*	8.3*	-2.7*	0.22±0.03		11.0±1.5^{X}		
**April 2018 - May 2020 (Time period 3)**													
A	Dike	7.0±0.3	0.5±0.1	1.0±0.06	22±0.6	89.5	-0.3±0.04	0.4±0.08	1.8±0.4		6.3±0.2	7.7	S2.3;S2.8;S2.11
B	Dike	8.7±0.3	3.5±0.7	1.6±0.1	26±0.6	89.5	-0.08±0.06	0.2±0.1	0.27±0.05		8.2±0.6	4.4	
	Mogi			0.5±0.07			1.6±0.05	0.6±0.05		3.2±0.2	5.8±0.4		
C	Dike	9.1 ±0.4	4.2±0.7	1.6±0.12	26±0.5	89.5	-0.1±0.07	0.1±0.13	0.25±0.04		9.3±0.7^{X}	3.9	
	Mogi			0.2±0.13			1.6±0.04	0.6±0.05		4.1±0.3	7.4±0.5^{X}		
	Decollement	10 km*	5 km*	10 km*	204.0*	5*	8.3*	-2.7*	0.27±0.04		13.5±2.0^{X}		

[Contd.]

Model	Source	Length (km)	Width (km)	Depth^{+} (km) asl	Strike (°)	Dip* (°)	Easting^{++} (km)	Northing++ (km)	Opening rate /slip rate (m/yr)	Vol. change rate (Δv/yr)	Potency rate (x10^{6} m3/yr)	RMSE (mm/yr)	Figs.
**January 2002- May 2005 (Time period A) [Data from[10]]**													
A	Dike	8.3±0.3	0.7±0.9	1.2±0.3	23.3±0.6	89.5*	-0.1±0.04	0.5±0.1	1.0±0.6		7.3±0.4	7.5	S2.4
B	Dike	8.5±0.3	3.3±0.6	1.7±0.1	25±0.6	89.5*	0.1±0.05	0.5±0.09	0.37±0.06		10.5±0.6	3.8	
	Mogi			0.0±0.08			1.4±0.07	0.9±0.08		2.3±0.1	4.1±0.2		
C	Dike	9.0±0.3	4.6±0.6	1.7±0.1	25±0.6	89.5*	0.1±0.07	0.5±0.1	0.3±0.03		12.3±0.8	3.6	
	Mogi			0.0±0.1			1.5±0.06	0.8±0.07		3.7±0.2	6.7±0.4		
	Decollement	10 km*	5 km*	10 km*	204*	5*	8.3*	-2.7*	0.24±0.03		12.3±1.6		
**January 2010 - January 2014 (Time period B)**													
D	Decollement	10*	5*	14*	204*	5*	6.1*	-7.1*	0.36±0.002		13.5	4.0	Fig. 2v

*($^{+}$): with respect to sea level, see text for impact of topography; (*): fixed parameters; (**),($^{++}$): center of upper edge of dislocation; ($^{+++}$): center of western edge of dislocation, (^{X}) used for Fig. 2. ^Source strength is expressed as potency, for dike/decollement it is (length * width * opening/slip)[56], for the mogi source it is 1.8*Δv[16] and for the fault slip is moment (length * width * slip).*

Table S2.2. *Parameters describing the prior probability distributions of the model parameters for the Bayesian inversion.*

**Source**	**Bounds**	**Length (km)**	**Width (km)**	**Depth^{+} (km)**	**Strike (°)**	**Dip* (°)**	**Easting^{++} (km)**	**Northing^{++} (km)**	**Opening/slip (m/yr)**	**Inflation (m^3)**
Dike	*Lower*	*5*	*0.5*	*2 (asl)*	*17*	*89.5**	*-10*	*-10*	*0.1*	
	Upper	*15*	*10*	*8 (bsl)*	*36*		*10*	*10*	*1.0*	
Mogi	*Lower*			*1 (asl)*			*-8*	*-8*		10^3
	Upper			*10 (bsl)*			*8*	*8*		10^9
Decollement	*Lower*	*10**	*5**	*10*(bsl)*	*204**	*5**	*0.01*	*-10*	*0.1*	
	Upper						*10*	*0.01*	*1.0*	

Table S2.3. *Depths obtained for different time periods when inverted for a dike with and without using adjustment suggested in Williams and Wadge (1998)*[18].

**Method**	**Inverted depth (m)**	**Reference elevation (m)**	**Depth a.s.l (m)**
**January 2014 - August 2015 (Time period 1)**			
depth-adjusted	2839	3666^{+}	827
depth-not-adjusted	2498	3325*	827
**August 2015 - April 2018 (Time periods 2)**			
depth-adjusted	2560	3643^{+}	1083
depth-not-adjusted	2856	3939*	1083
**April 2018 - May 2020 (Time periods 3)**			
depth-adjusted	2723	3699^{+}	976
depth-not-adjusted	2918	3894*	976

($^{+}$) Mean of all data points. (*) Calculated such that the depth a.s.l. is the same for both approaches.

Table S2.4. *Potency of the magma sources for the 5 periods together with the equivalent widening, excess pressure, and decollement slip for the 5 periods. Both rates and total change are shown for each period.*

**Time period**	**opening dislocation potency [10^6 m^3/yr] (total [10^6 m^3])**	**mogi potency [10^6 m^3/yr] (total [10^6 m^3])**	**dike-like magma body potency [10^6 m^3/yr] (total [10^6 m^3])**	**equivalent widening [cm/yr] (total [cm])**		**excess pressure[xxx] [MPa/yr] (total MPa)**		**decollement slip* [cm/yr] (total [cm]), M_w****	**comments**
				**dike dimensions from table S2.1**	**dike dimension 9x5 km^2 +**	**dike widths from table S2.1 (varying crack-size model)**	**dike width 5 km (uniform crack-size model)**		
2002-2006	12.3(45.1)	6.7(24.6)	19.0(69.7)	46(168)	42 (155)	2.7(10.1)	3.6(13.2)	24(88.0), M_w5.9	See Table S2.1
Jan. 2006 - Jan. 2010	-	-	4.7(18.8)[x]	11.5(42)[x]	10.4(42)[x]	0.7(2.5)[x]	0.9(3.6)[x]	6(24.0)[x], M_w5.5	rough estimate
Jan. 2010 - Jan. 2014	0	0	0	0	0	0	0	33 (134.7)[xx], M_w6.0	See Table S2.1
Jan. 2014 - Aug. 2015	20.0(31.7)	8.1(12.8)	28.1(44.5)	40.3(63.8)	62.4 (98)	2.0(3.2)	5.3(8.4)	21 (33.3), M_w5.6	See Table S2.1
Aug. 2015 - Apr. 2018	6.4(17.1)	5.4(14.4)	11.8(31.5)	37.6(100.4)	26.2 (70)	5.3(13.3)	2.2(6.0)	22 (58.8), M_w5.75	See Table S2.1
Apr. 2018 - May 2020	9.3(19.4)	7.4(15.4)	16.7(34.8)	43.6(91.0)	37.1 (77)	4.1(8.3)	3.2(6.6)	27 (56.3), M_w5.74	See Table S2.1
Total 2014-2020	68.2x10^6 m^3	42.6	110.8	255	245	24.8	21	148 m, M_w6.0	
Total 2002-2020	113.3	67.2	199.3	465	442	37.4	38	3.95 m, M_w6.3	

(+) widening calculated using $\frac{dike\ potency + mogi\ potency}{9\ km*5\ km}$, *(*) on 10*5 km^2 fault, (**) calculated using M_w=⅔ $log_{10}(\mu M_0)$-6.03 with μ the rigidity (16 x 10^9 Pa) and M_0 the geometric moment (m^3), (X) 25 % of 2002-2005 opening estimated from data shown in Fig. 4 of Pepe et al (2018)[56]. (XX) using 2010-2014 rate. (XXX) calculated using equation in (3.1) with a Young's modulus E of 40 x 10^9 Pa.*

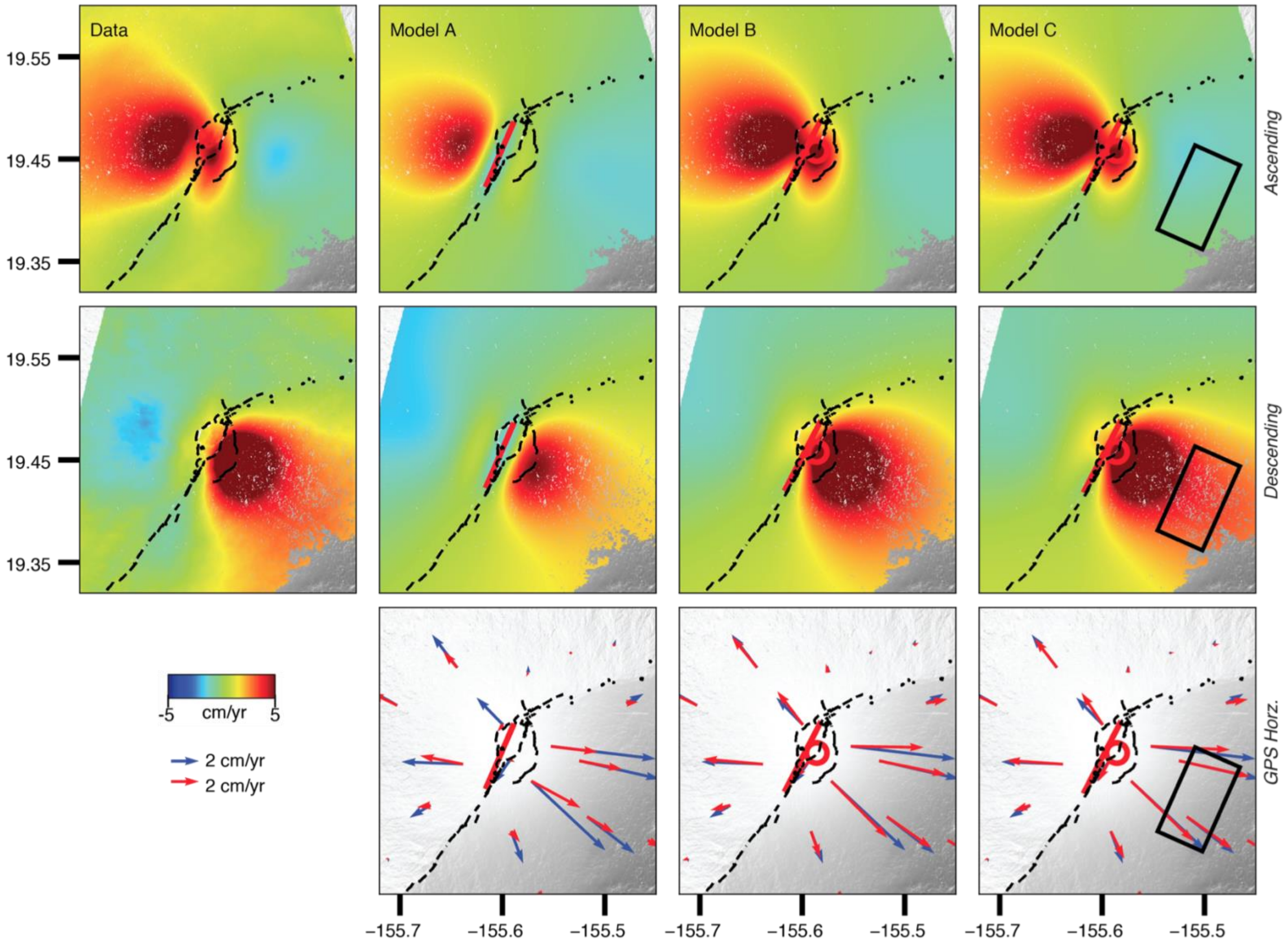

Figure S2.1. *Data-model comparison for February 2014 - August 2015 using InSAR [Ascending, Descending] and GPS horizontal*

velocities (blue vectors: data; red vectors: model). Columns 2,3 and 4 show the model predictions for models A, B and C of table S2.1, respectively.

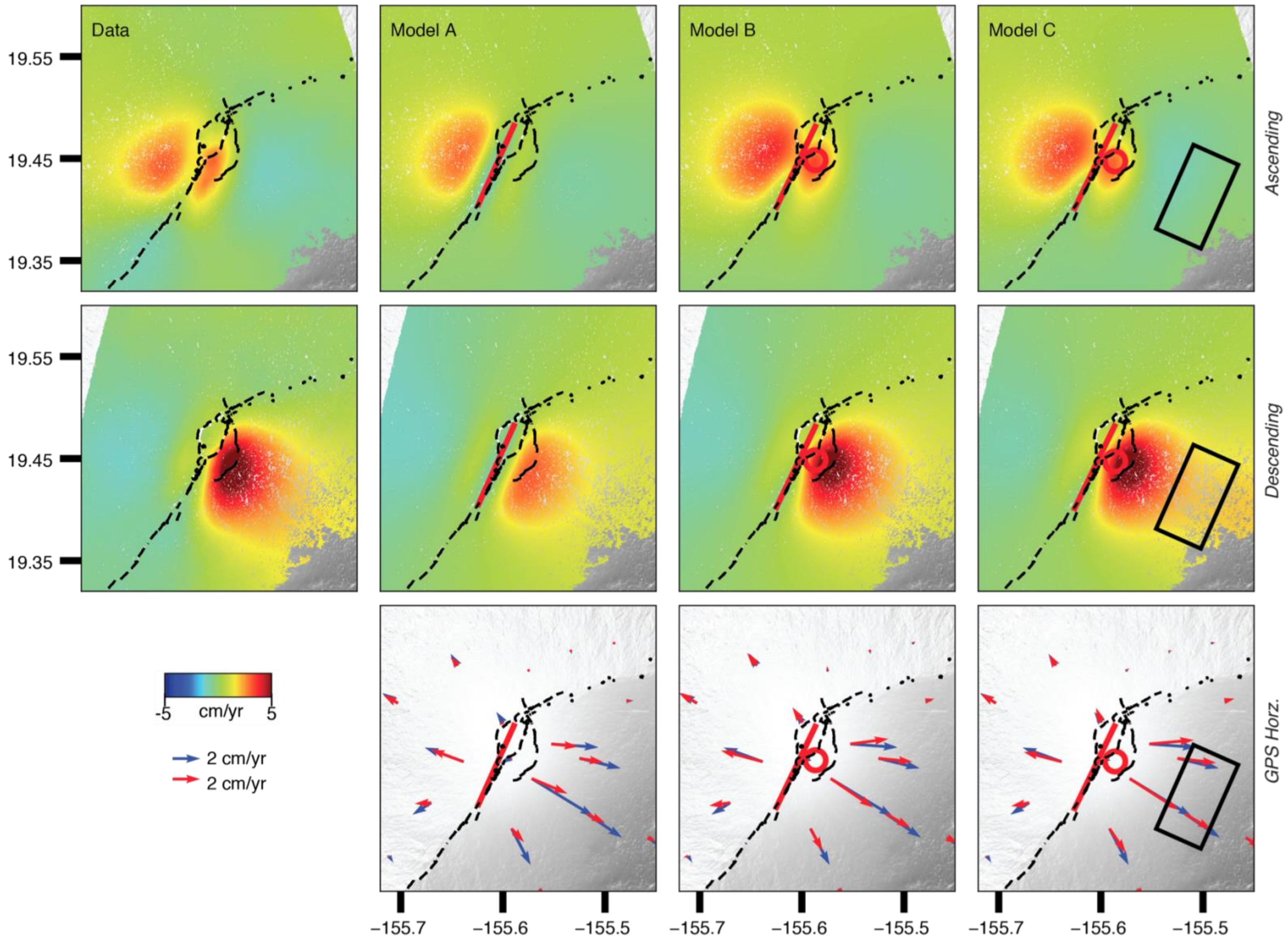

Figure S2.2. *Same as Fig. S2.1 for August 2015 - April 2018.*

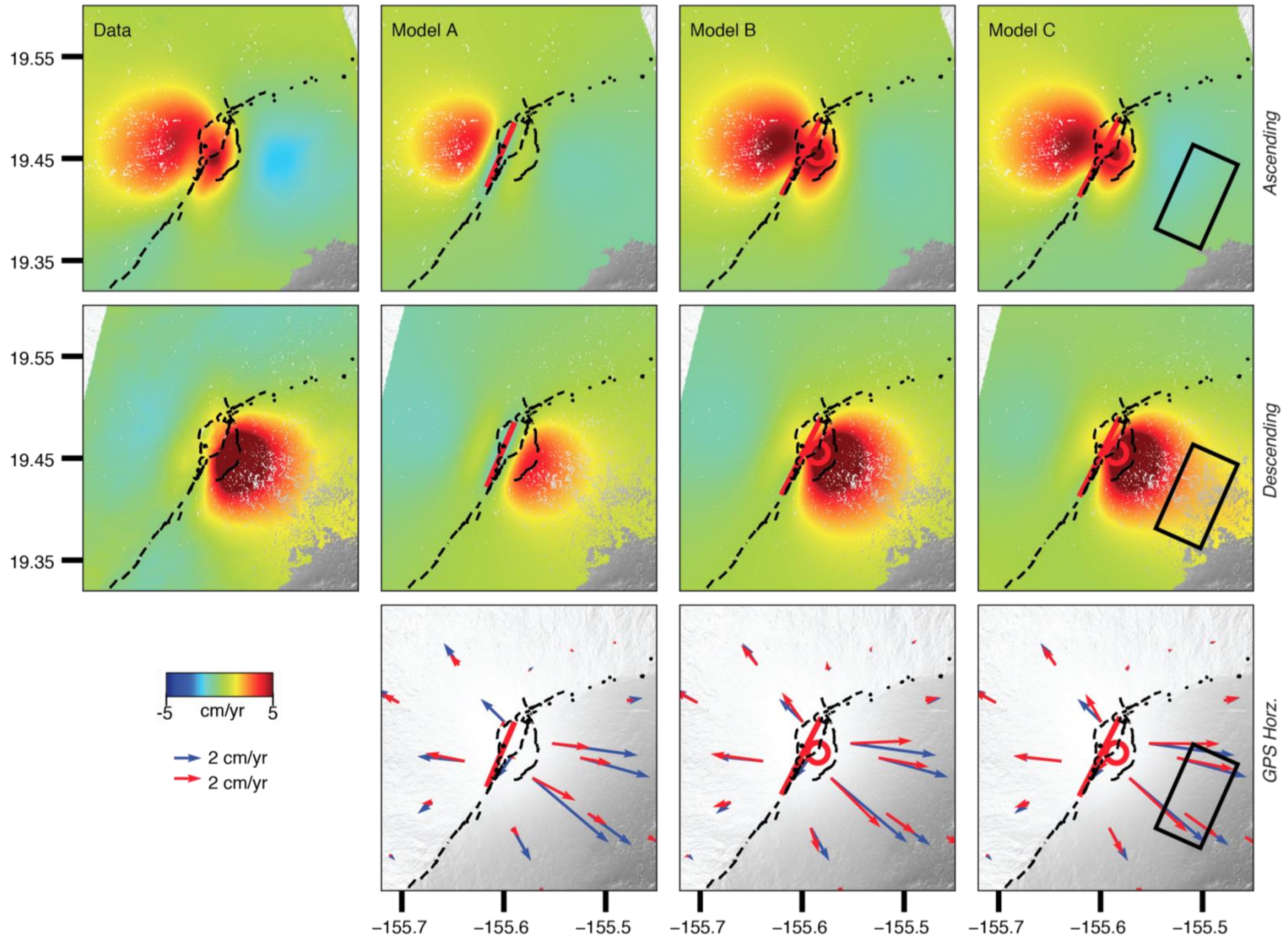

Figure S2.3. *Same as Fig. S2.1 for April 2018 - May 2020.*

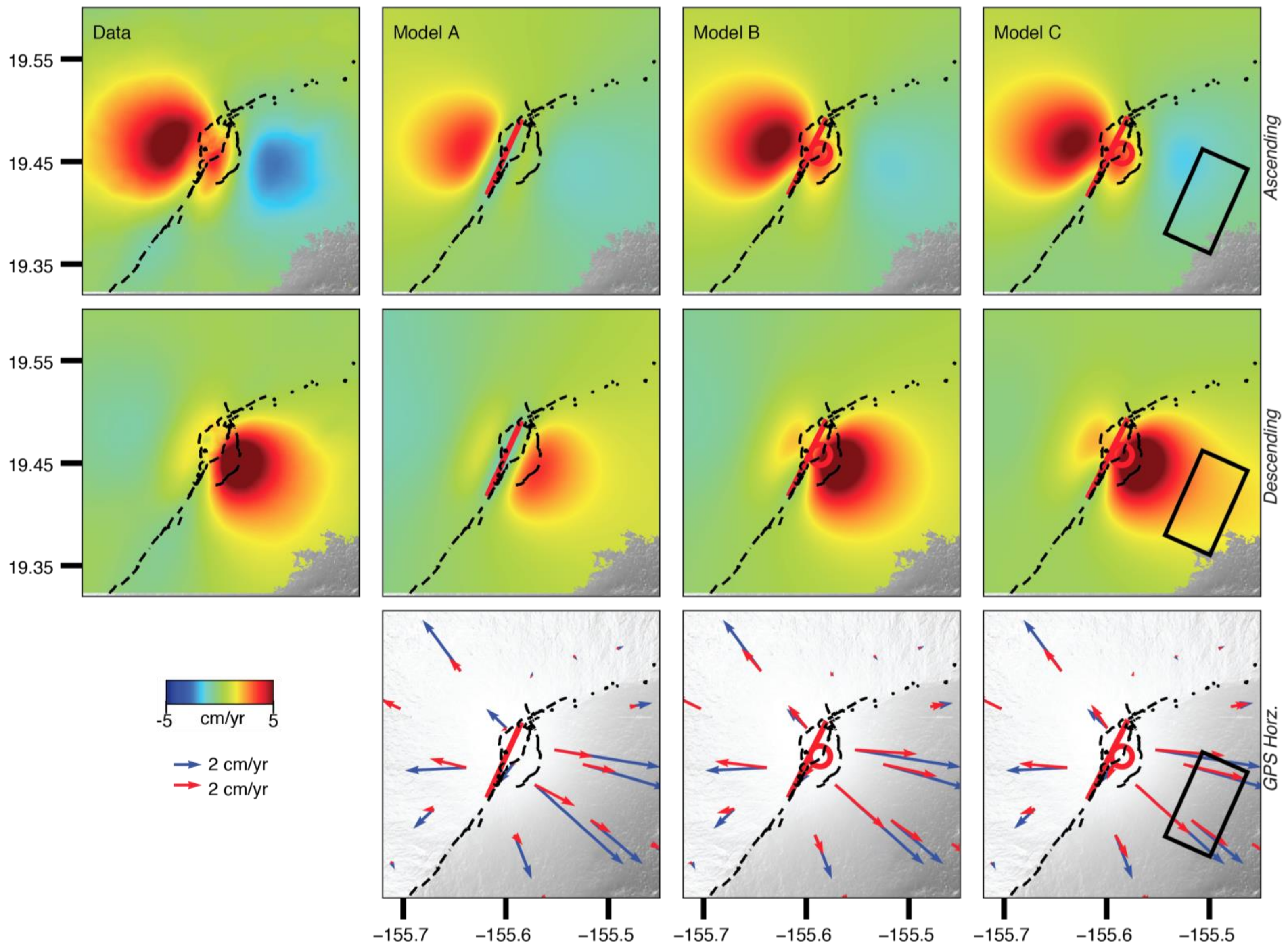

Figure S2.4. *Same as Fig. S2.1 for 2002-2005 using data from Amelung et al.*[10].

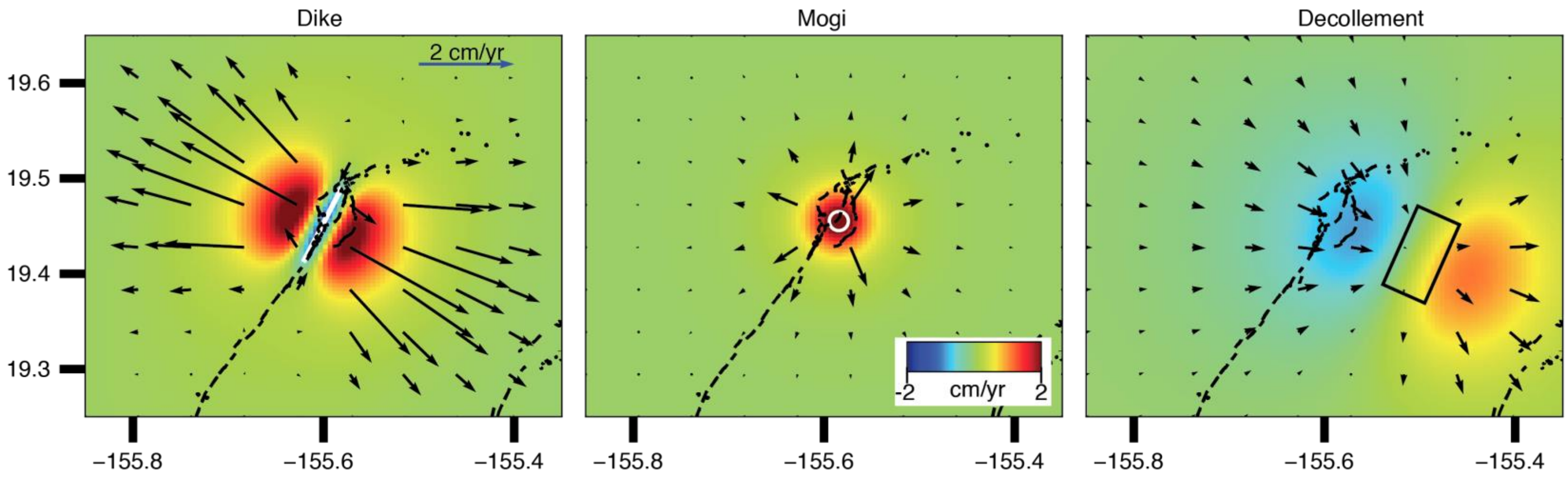

Figure S2.5. *Surface velocities from (a) opening dislocation, (b) Mogi source and (c) decollement slip of the 2018-2020 model (0.25 cm/yr dislocation opening, 27 cm/yr decollement slip), illustrating the difficulties for separating the contributions from dike widening and decollement slip to the observations. Vectors: horizontal velocities; color shading: uplift rate. Note that 27 cm/yr decollement slip cause only 1 cm/yr surface velocities.*

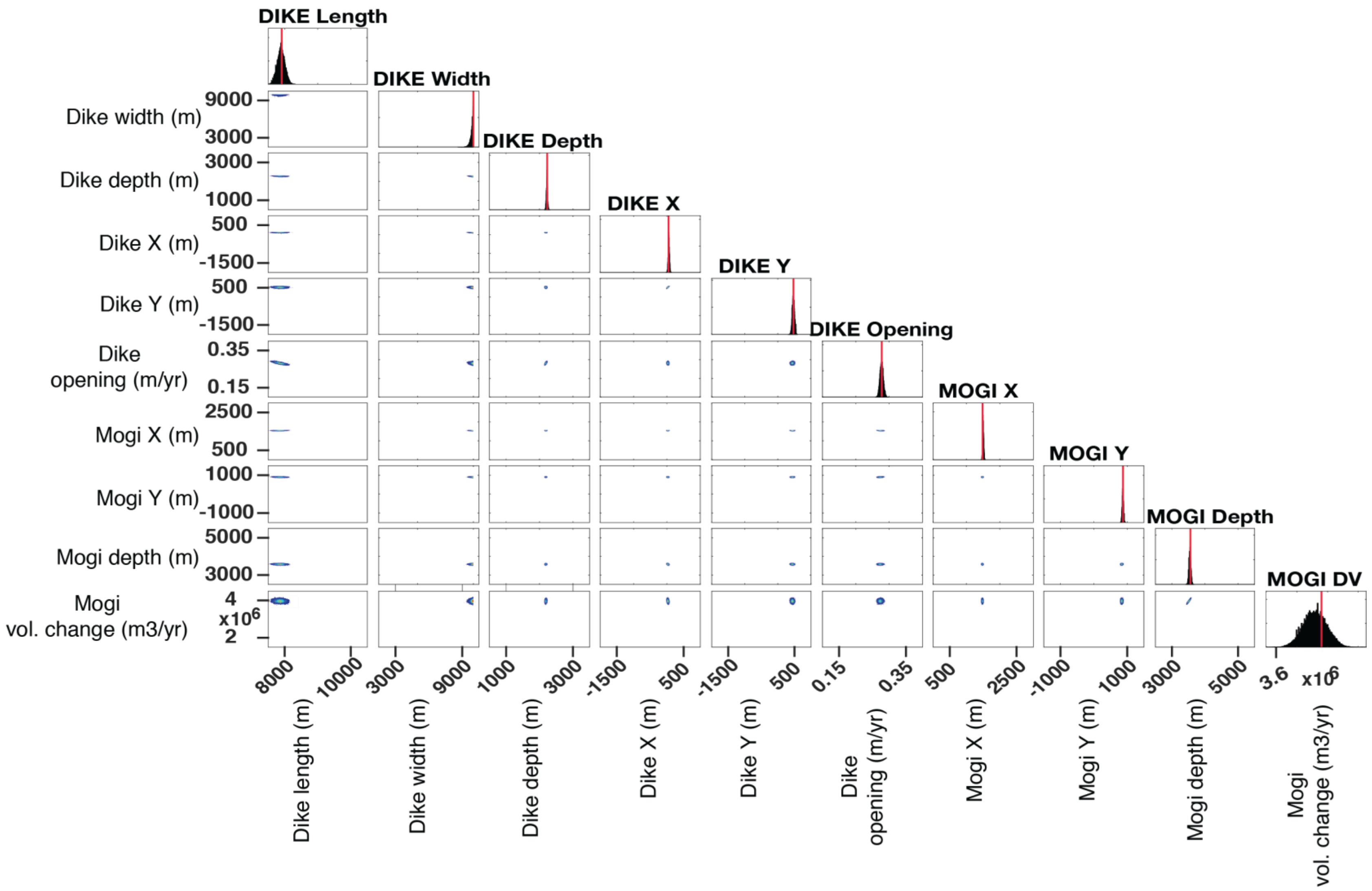

Figure S2.6. *Joint probability distribution function plots between different parameters used in the inversion for February 2014-August 2015 (Time period 1) and configuration of model B.*

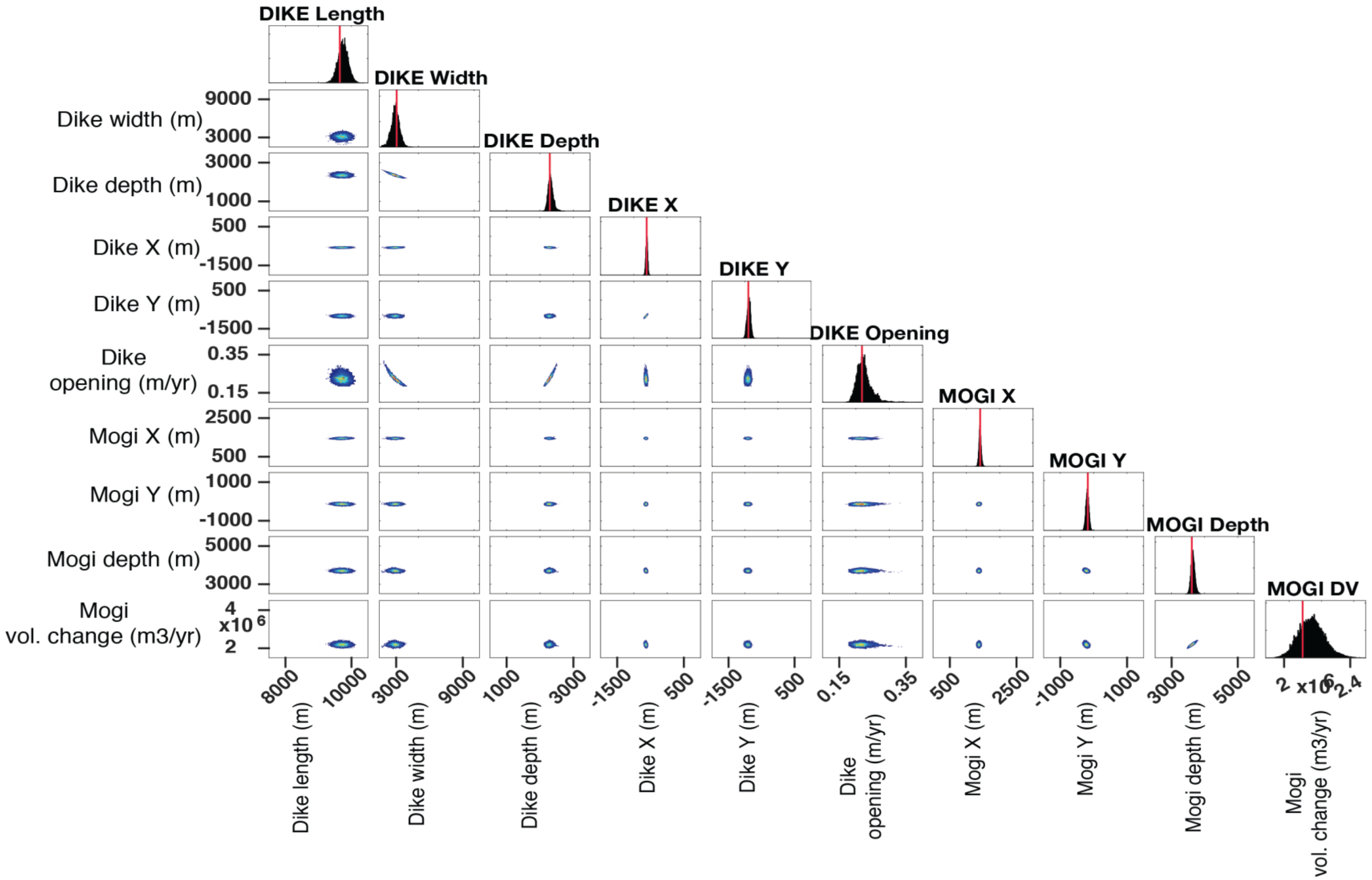

Figure S2.7. *Same as Fig. S2.6 for period August 2015 - April 2018 (Time period 2).*

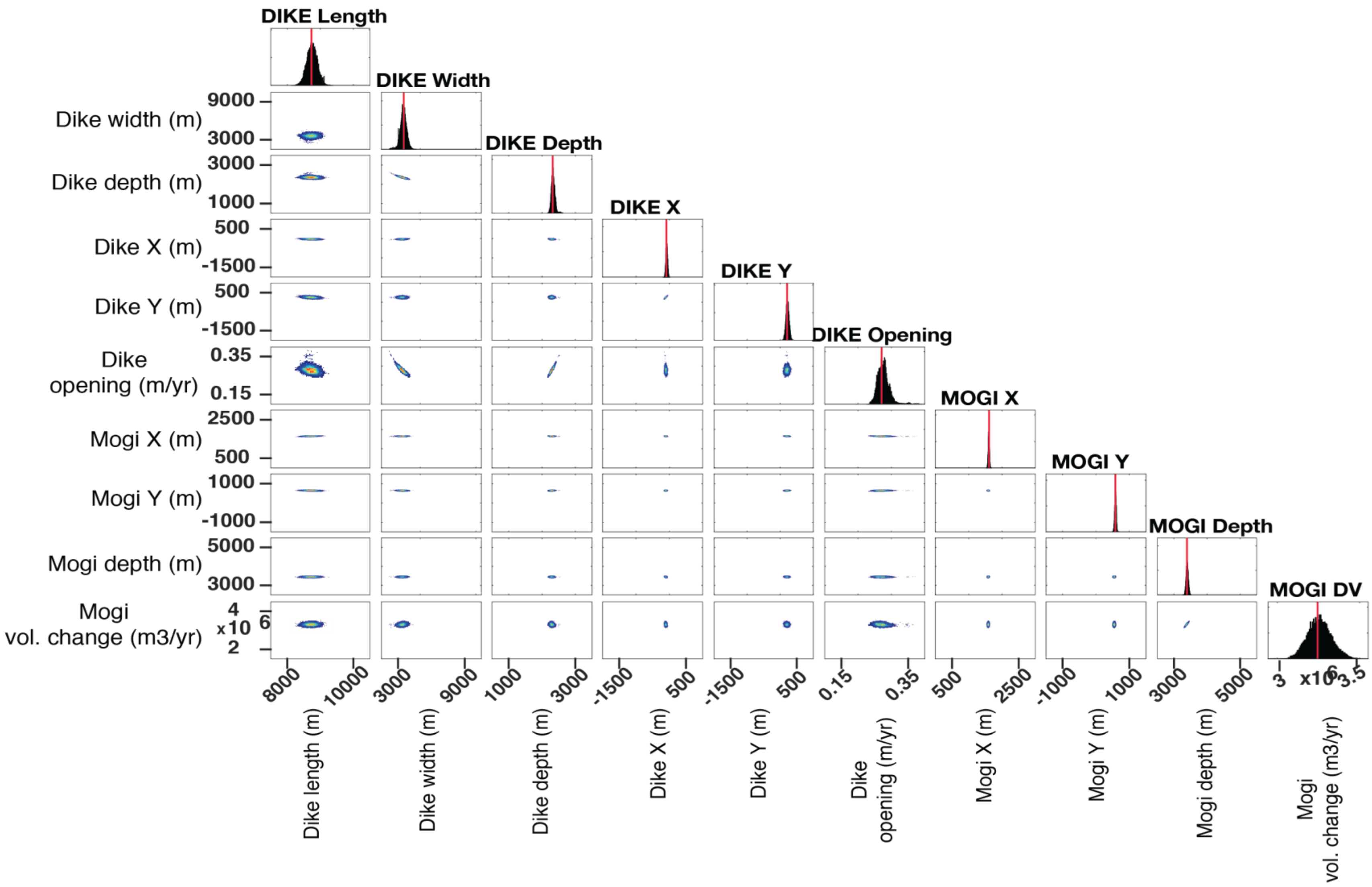

Figure S2.8. *Same as Fig. S2.6 for period April 2018 - May 2020 (Time period 3).*

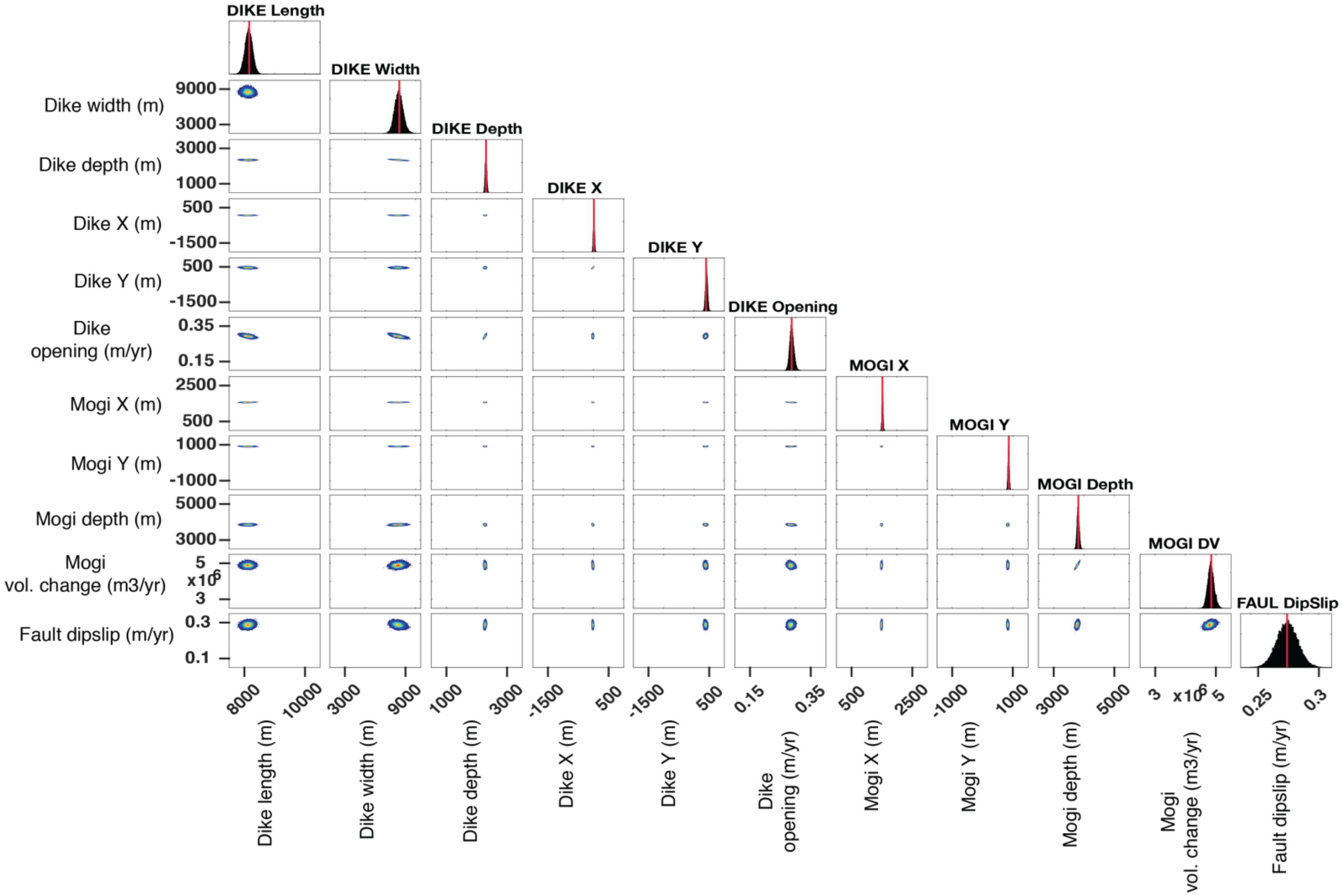

Figure S2.9. *Joint probability distribution function plots between different parameters used in the inversion for period Feb. 2014-Aug. 2015 (Time period 1) and configuration of model C.*

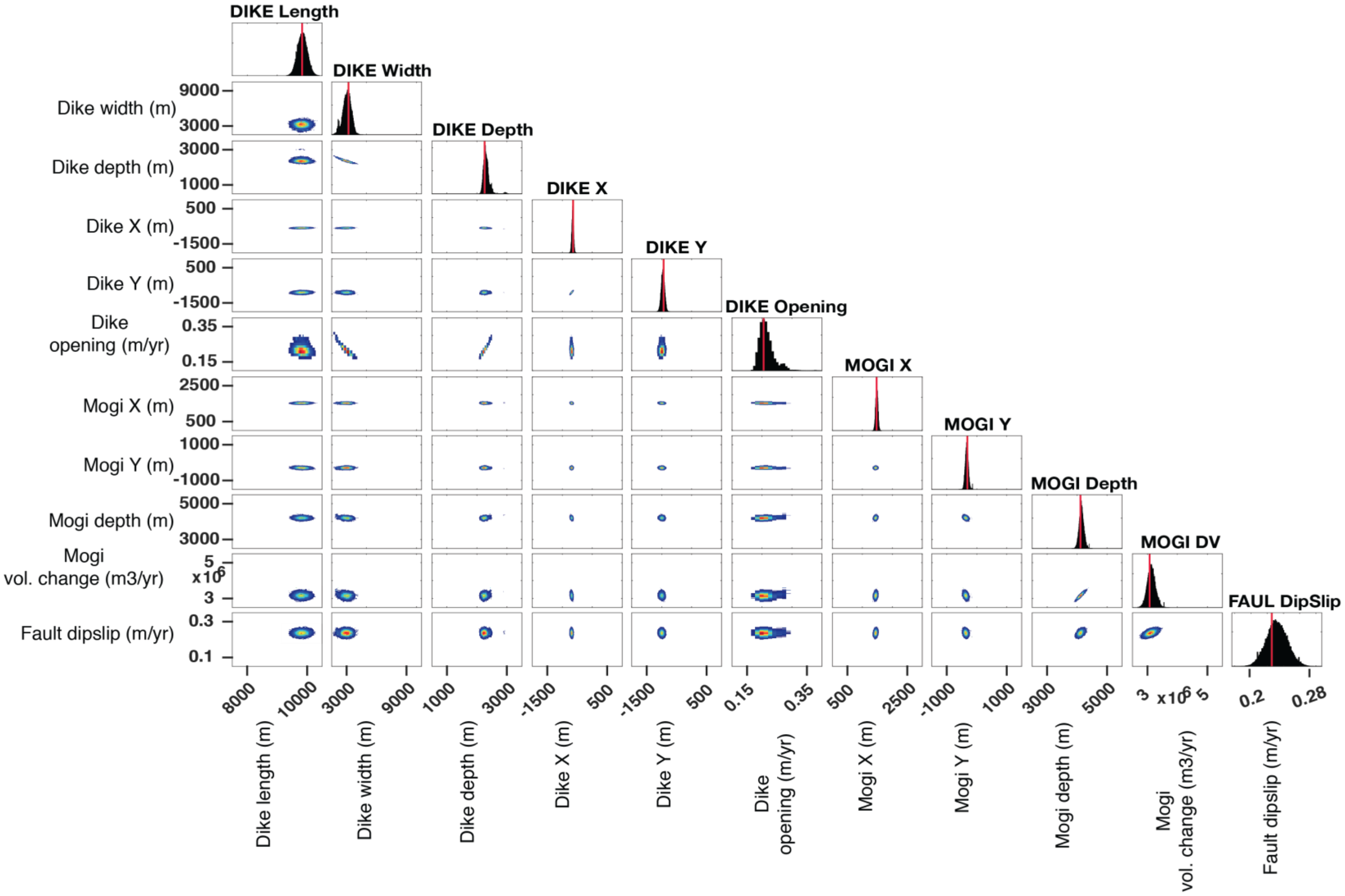

__Figure S2.10.__ Same as Fig. S2.9 for August 2015 - April 2018 (Time period 2).

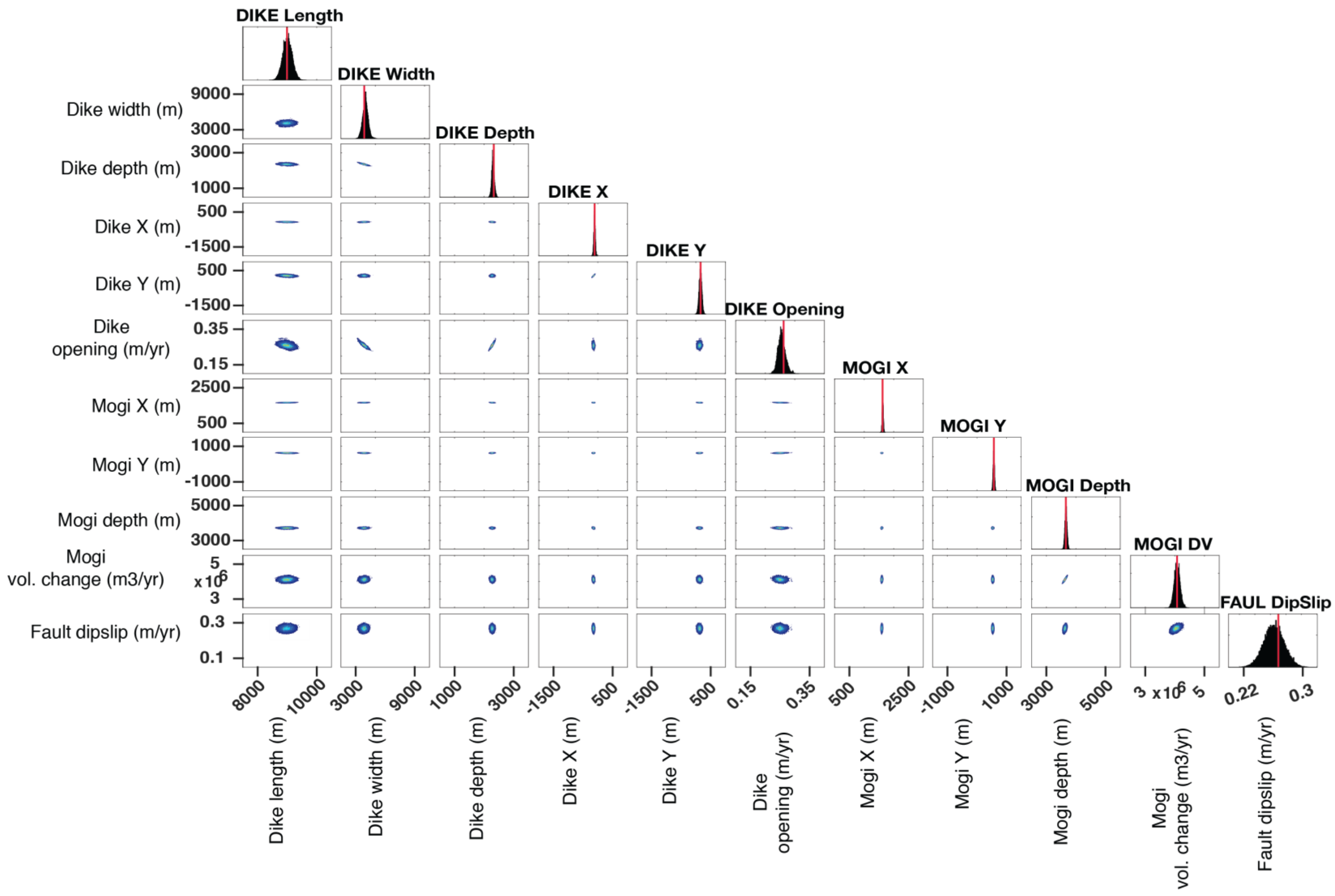

Fig. S2.11. *Same as Fig. S2.9 for period April 2018 - May 2020 (Time period 3).*

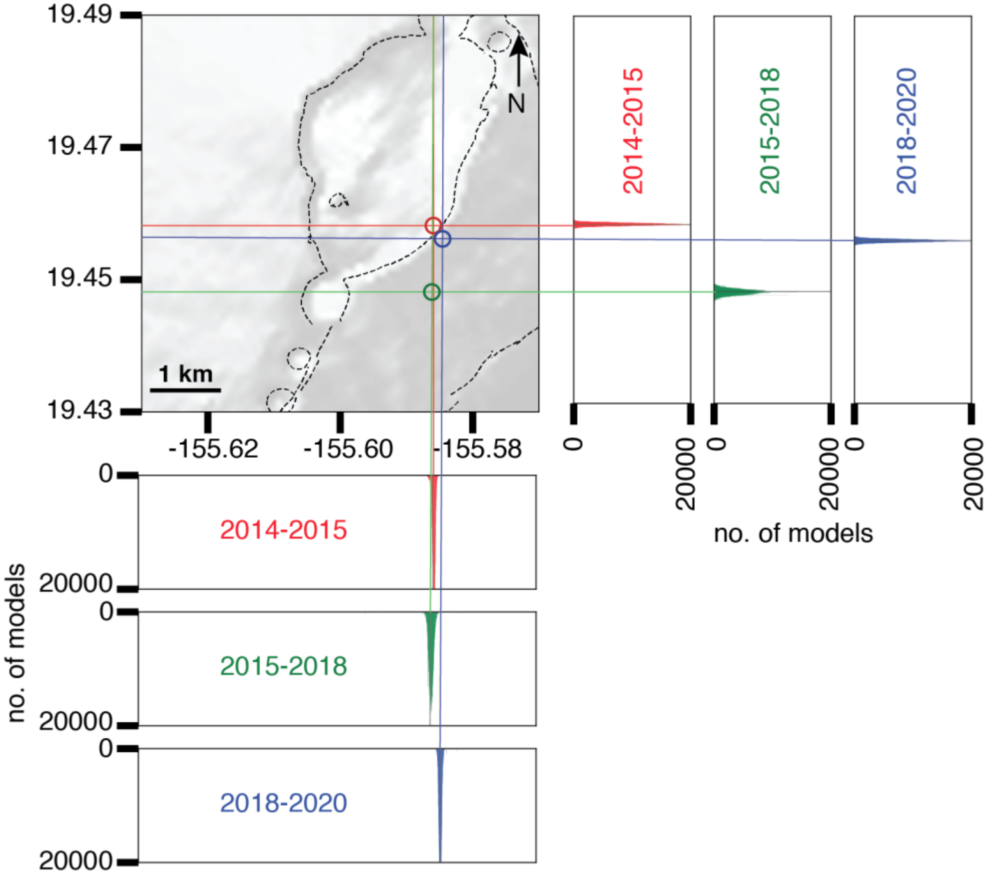

Figure S2.12. *Density distribution of spatial location (easting, northing) of mogi source around the summit caldera from the inversion of three time periods.*

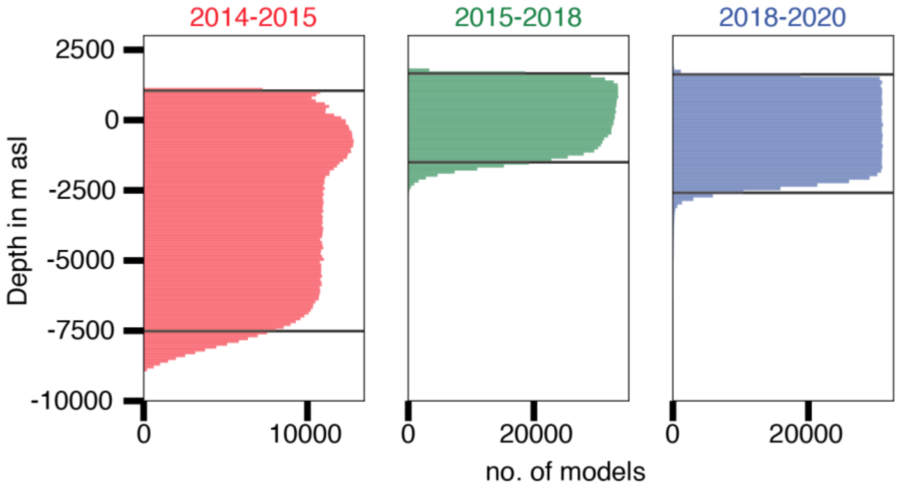

Figure S2.13. *Density distributions of dike extent (depth-wise) from the model inversions*.

S3. Stress change modelling

S3.1: Finite element method

To calculate the stress field under Mauna Loa, we built a Finite Element Model (FEM) as shown in Fig. S3.1. The topography of the Big Island is built into the model using SRTM dem (90 m). Boundary conditions include roller supports on all sides and fixed boundary at the bottom (Fig. S3.1a) following [57,58]. The top surface is let free to deform in all directions. The mesh for the computation is fine near the sources of deformation and coarsens towards the outer boundaries (Fig. S3.1b). Total size of the model is 300 km x 300 km x 300 km consisting of >100000 triangular elements. The size of the model is big enough in all directions so that calculated displacements are not affected by the boundaries. The section for the calculations is taken on a line passing through the modelled dike on the rift zone as indicated in (Fig. S3.1c). As described in the main paper, we compute perturbations to the lithostatic stress state under Mauna Loa due to, (i) magma body pressurization, (ii) decollement slip and (iii) topographic load.

We modelled our magmatic sources as lens shaped cavities in an isotropic medium and its perturbation to the lithostatic stress (magma body widening) is applied to the cavity walls as constant excess pressure (P_e),

$$P_e = \frac{b * E}{2 * (1 - v^2) * L}$$

where, b is the widening of the dike, E is Young's modulus and L is half of the dip-dimension of the dike. v is Poisson's ratio.

Since our best fitting model is a complex magma body described by a combination of an opening dislocation and point source, we used the sum of dike and mogi potencies to compute equivalent widening (b) on a 9 * 5 km^2 dislocation. The thickness of the lens shaped cavity is kept at 50 m, previous studies have shown that this choice won't have a significant influence on the final result[58].

We modelled the decollement as a 10*5 km^2 shaped rectangular cavity and a slip is applied to the walls. Topographic load is applied as body load on top of a lithostatic crust corresponding to ρgh (ρ is the density; g is the acceleration due to gravity; h is the elevation above 1.7 km a.s.l.).

Stress perturbations are computed on a section passing through the magma body along the rift zone as shown in Fig. 3.1c.

S3.2: Elastic properties from seismic tomography

For the computations, we assume the material is a homogeneous isotropic linear elastic solid with density (ρ) of 2800 kg/m^3. Other elastic parameters are obtained from seismic velocities (V_p ,V_s) under Mauna Loa[59] (see Fig. S3.2). V_p and V_s are related to the Lame's constants (μ and λ) by,

$$\mu = \rho * V_s^2 ; \lambda = \rho * V_p^2 - 2\mu;$$

From which, we derive Poisson's ratio (ν), Bulk modulus (K), Young's Modulus (E) and Shear Modulus (G) as,

$$\nu = \frac{\lambda}{2*(\lambda+\mu)} ; K = \lambda + \frac{2\mu}{3} ; E = \frac{9K\lambda}{3K+\mu} ; G = \frac{E}{2*(1+\nu)} ;$$

For, V_p of 4.25 m/s and (V_p / V_s) of 1.73, we obtain ν = 0.25, K = 27 GPa, E = 40 GPa and G = 16 GPa.

S3.3 Analytical and FEM models comparison:

To validate our FEM results, a comparison is made using analytical dislocations[48] for 1m opening over a dike (Fig. S3.3a) and 1m dip slip over a thrust fault (Fig. S3.3b).

S3.4: Stress concentrations arising around the magma body

In Fig. 4a, c we show the rift-perpendicular normal stress perturbations along a section passing through the rift zone due to decollement slip and the topographic load, calculated assuming an isotropic elastic material. However, if the magma body is considered a fluid-filled cavity that deforms in response to imposed stress, it leads to stress concentrations along the boundaries[60]. The normal stress perturbations with cavity present are shown in Fig. S3.4a,c. For the cavity we used lens-shaped dike with dimensions of dislocation from time period 1 and maximum thickness of 50 m at center, gradually reducing to 0 m at edges. The choice of the cavity thickness does not significantly affect the stresses produced[58]). However, stress concentrations near at edges of the cavity are ~50% higher when compared to without cavity.

S3.5: Earthquake potential along decollement fault

To evaluate the effect of inflation of the dike-like magma body on decollement slip, we considered three different-sized faults (10*5 km^2, 20*20 km^2 , 30*30 km^2 see Fig. S3.5), divided them into 1*1 km^2 patches, and used the finite element model to calculate

the slip in response to the imparted magma body widening (Table S3.5.1). We calculate a total moment (M_0) in Nm and Moment magnitude[61] (M_w) as:

$$M_0 = G * Area * slip$$

$$M_w = \frac{2}{3}\log(M_0) - 6.03$$

A summary of moment imparted over the decollement due to magma body widening since 2002, is given in table S3.5.2.

S3.6 Tensional stress due to magma body widening in the rift zone:

Tensional stress due to magma body widening from three time periods (Fig. S3.6) used in the modelling and cumulative (Fig. 4d) accumulated so far in the rift zone has been calculated. Tensional stress in the rift zone accumulates as the magma body widens and gets relieved in the zone where the magma body intrudes[29].

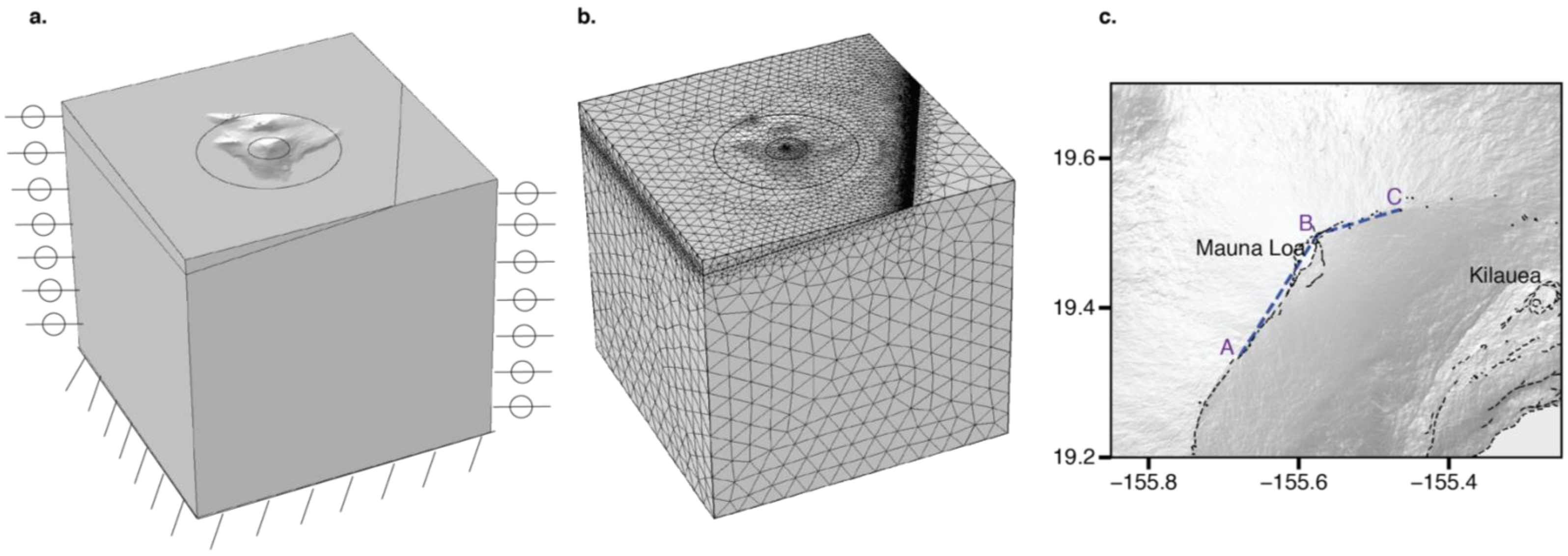

Figure S3.1. *a. COMSOL model generated, indicating the boundaries; b. Mesh showing varying lengths and key boundaries; c. Section used for evaluating normal stress.*

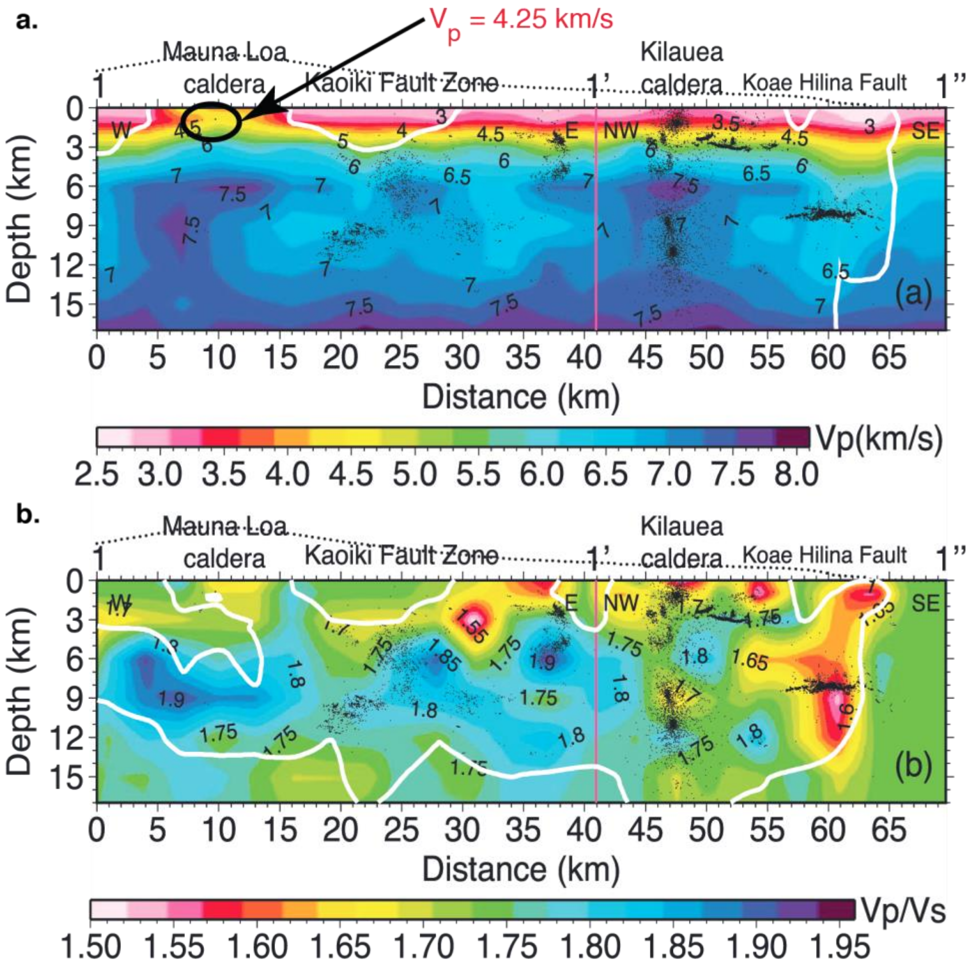

Figure S3.2. *Seismic velocities under Mauna Loa and Kilauea from Fig. 13 of (59) showing a.* V_p *and b.* V_p/V_s *ratio. Black circle indicates the region used to obtain velocities for the current paper.*

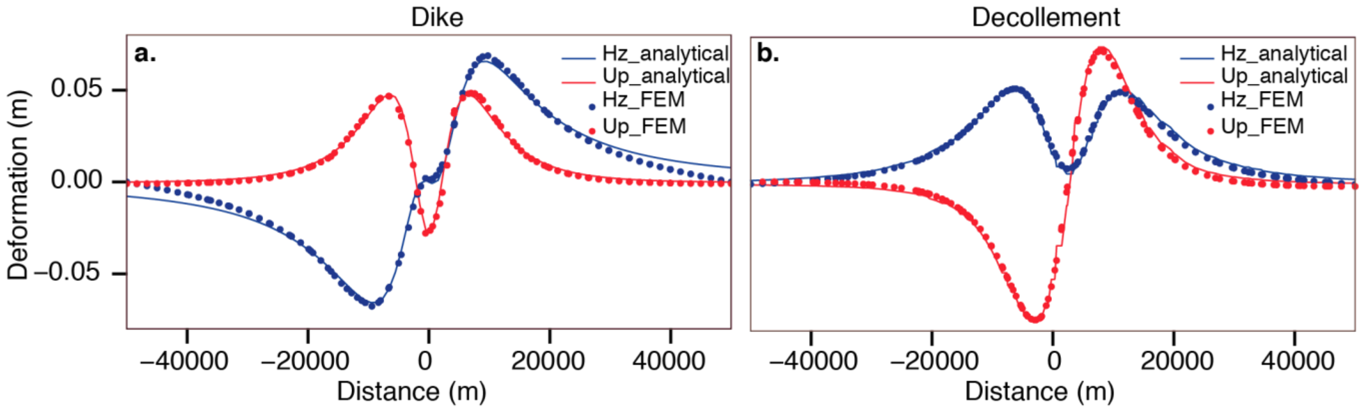

Figure S3.3. *Comparison between horizontal (Hz) and vertical (Up) deformation calculated using the analytical solution (solid lines) and FEM (circles) for (a) a 10*5 km^2 dike with 1 m opening and (b) 10*5 km^2 thrust fault slipping 1 m in dip direction.*

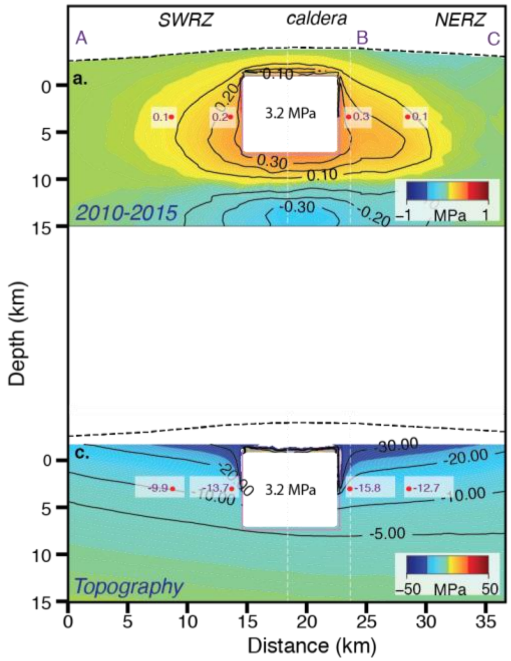

Figure S3.4. *Similar to Fig. 3a,c but with cavity present.*

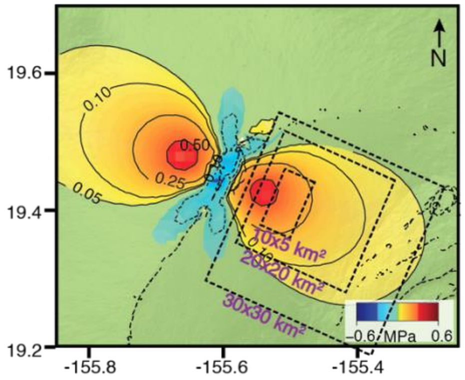

Figure S3.5. *Black rectangles are fault patches considered in Table S3.2.1. Background is the same as Fig. 3e.*

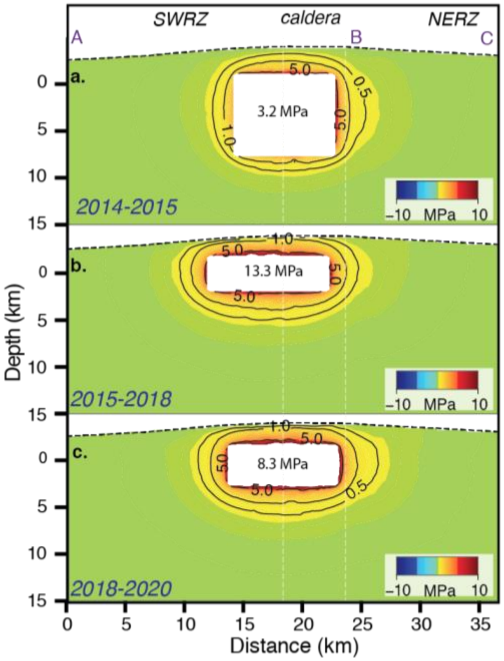

Figure S3.6. *Same as Fig. 3b, for three individual time periods, a. 2014-2015; b. 2015-2018; C. 2018-2020.*

Table S3.5.1: *Slip measured on decollement with different fault sizes.*

**Source/ Widening on 9x5 km^2 dike**	**Size of Fault [km^2]**	**Average slip [m]**	**Total slip [m]**	**Geometric moment M_0 (Potency) [Nm]**	**M_w (**)**
1m	10 x 5	0.02	1.3	$16\text{x}10^9$x1000x1000x1.30	4.84
1m	20 x 20	0.02	9.89	$16\text{x}10^9$x1000x1000x9.89	5.43
1m	30 x 30	0.02	38.36	$16\text{x}10^9$x1000x1000x38.36	5.82
5.0 m	10x5	0.112	6.5	$16\text{x}10^9$x1000x1000x7.28	5.35
5.0 m	20 x 20	0.112	49.4	$16\text{x}10^9$x1000x1000x55.4	5.90
5.0 m	30 x 30	0.112	193.15	$16\text{x}10^9$x1000x1000x193.15	6.30

for a 1m slip on 10 km x 5 km Mw is 5.9, (**) calculated using $M_w = \frac{2}{3}\log(M_0) - 6.03$ *for 0.3 m slip on 10*5 km*^{2} M_w *is 5.55.*

Table S3.5.2: *Slip measured on decollement due to magma body from three time periods.*

**Time period**	**Dike dimension [km^2]**	**Widening**	**Size of Fault [km^2]**	**Average slip [m]**	**Total slip-on fault patches [m]**	**Geometric moment M_0 (Potency) [Nm]**	**M_w (**)**
2002-2006	9x5	1.5	20 x 20	0.02	16.8	16x10^{9}x1000x1000x16.8	5.59
2006-2010	9x5	0.4	20 x 20	0.02	4.9	16x10^{9}x1000x1000x4.9	5.19
2014-2015	9x5	1.0	20 x 20	0.02	10.9	16x10^{9}x1000x1000x10.9	5.46
2015-2018	9x5	0.7	20 x 20	0.02	7.9	16x10^{9}x1000x1000x7.9	5.37
2018-2020	9x5	0.8	20 x 20	0.02	8.9	16x10^{9}x1000x1000x8.9	5.40
Total imparted		4.4			49.4	16x10^{9}x1000x1000x49.4	5.90

(**) calculated using $M_w = \frac{2}{3}\log(M_0) - 6.03$.

References:

10. Amelung, F., Yun, S. H., Walter, T. R., Segall, P. & Kim, S.W. Stress control of deep rift intrusion at Mauna Loa volcano, Hawaii. *Science* **316**, 1026-1030 (2007).

16. Bonafede, M. & Ferrari, C. Analytical models of deformation and residual gravity changes due to a Mogi source in a viscoelastic medium. *Tectonophysics* **471**, 4-13 (2009).

18. Williams, C.A. & Wadge, G. The effects of topography on magma chamber deformation models: Application to Mt. Etna and radar interferometry. *Geophysical Research Letters* **25**, 1549-1552 (1998).

29. Rivalta, E. *et al*. Stress inversions to forecast magma pathways and eruptive vent location. *Science advances* **5**, eaau9784 (2019).

44. Yunjun, Z., Fattahi, H. & Amelung, F. Small baseline InSAR time series analysis: Unwrapping error correction and noise reduction. *Computers & Geosciences* **133**, 104331 (2019).

45. Fattahi, H. & Amelung, F. DEM error correction in InSAR time series. *IEEE Transactions on Geoscience and Remote Sensing* **51**, 4249-4259 (2013).

46. Doin, M. P., Lasserre, C., Peltzer, G., Cavalié, O. & Doubre, C. Corrections of stratified tropospheric delays in SAR interferometry: Validation with global atmospheric models. *Journal of Applied Geophysics* **69**, 35-50 (2009).

47. Blewitt, G., Hammond, W.C. & Kreemer, C. Harnessing the GPS data explosion for interdisciplinary science. *Eos* **99**, 1-2 (2018).

48. Okada, Y. Internal deformation due to shear and tensile faults in a half-space. *Bulletin of the seismological society of America* **82**, 1018-1040 (1992).

49. Mogi, K. Relations between the eruptions of various volcanoes and the deformation of the ground surfaces around them. *Bulletin of the Earthquake Research Institute University of Tokyo* **36**, 99-134 (1958).

50. Decriem, J. *et al*. The 2008 May 29 earthquake doublet in SW Iceland. *Geophysical Journal International* **181**, 1128–1146 (2010).

51. Lohman, R. B. & Simons, M. Some thoughts on the use of InSAR data to constrain models of surface deformation: Noise structure and data downsampling. *Geochemistry*, *Geophysics*, *Geosystems* **6**, Q01007 (2005).

52. Bagnardi, M. & Hooper, A. Inversion of surface deformation data for rapid estimates of source parameters and uncertainties: A Bayesian approach. *Geochemistry, Geophysics, Geosystems* **19**, 2194-2211 (2018).

53. Nikkhoo, M., Walter, T. R., Lundgren, P. R. & Prats-Iraola, P. Compound dislocation models (CDMs) for volcano deformation analyses. *Geophysical Journal International* **208**, 877–894 (2017).

54. Lundgren, P. *et al*. Source model for the Copahue volcano magma plumbing system constrained by InSAR surface deformation observations. *Journal of Geophysical Research: Solid Earth* **122**, 5729-5747 (2017).

55. Aki, K. & Richards, P. G. Quantitative Seismology (University Science Books, 2002).

56. Pepe, S. *et al*. The use of massive deformation datasets for the analysis of spatial and temporal evolution of Mauna Loa volcano (Hawai'i). *Remote Sensing* **10**, 968 (2018).

57. Grosfils, E. B. *et al*. Elastic models of magma reservoir mechanics: a key tool for investigating planetary volcanism, *Geological Society London Special Publications* **401**, 239-267 (2013).

58. Albino, F., Biggs, J. & Syahbana, D. K. Dyke intrusion between neighbouring arc volcanoes responsible for 2017 pre-eruptive seismic swarm at Agung. *Nature Communications* **10**, 748 (2019).

59. Lin, G., Shearer, P. M., Matoza, R. S., Okubo, P. G. & Amelung, F. Three-dimensional seismic velocity structure of Mauna Loa and Kilauea volcanoes in Hawaii from local seismic tomography. *Journal of Geophysical Research: Solid Earth* **119**, 4377-4392 (2014).

60. Gudmundsson, A. Magma chambers: Formation, local stresses, excess pressures, and compartments. *Journal of Volcanology and Geothermal Research* **237**, 19–41 (2012).

61. Hanks, T.C. & Kanamori, H. A moment magnitude scale. *Journal of Geophysical Research: Solid Earth* **84**, 2348-2350 (1979).
